# Apolipoprotein E mimetic peptide COG1410 combats pandrug-resistant *Acinetobacter baumannii*

**DOI:** 10.3389/fmicb.2022.934765

**Published:** 2022-08-23

**Authors:** Bo Wang, Feng-Wan Zhang, Wei-Xiao Wang, Yan-Yan Zhao, Su-Yue Sun, Jin-Hong Yu, Michael P. Vitek, George F. Li, Rui Ma, Shiwei Wang, Zhiliang Hu, Wei Chen

**Affiliations:** ^1^Institute of Medicinal Biotechnology, Chinese Academy of Medical Sciences and Peking Union Medical College, Beijing, China; ^2^Department of Infectious Disease, The Second Hospital of Nanjing, Nanjing University of Chinese Medicine, Nanjing, China; ^3^Clinical Research Center, The Second Hospital of Nanjing, Nanjing University of Chinese Medicine, Nanjing, China; ^4^Department of Clinical Laboratory, The Second Hospital of Nanjing, Nanjing University of Chinese Medicine, Nanjing, China; ^5^Cognosci, Inc., Durham, NC, United States; ^6^Shanghai Nanoport, Thermofisher Scientific, Shanghai, China; ^7^Key Laboratory of Resources Biology and Biotechnology in Western China, Ministry of Education, College of Life Science, Northwest University, Xi'an, China; ^8^Center for Global Health, School of Public Health, Nanjing Medical University, Nanjing, China

**Keywords:** *Acinetobacter baumanii*, pandrug-resistant, antimicrobial peptide, ApoE, COG1410, synergistic interaction, mechanism of action

## Abstract

The emergence of pandrug-resistant bacteria breaks through the last line of defense and raises fear among people of incurable infections. In the post-antibiotic era, the pharmaceutical field turns to seek non-conventional anti-infective agents. Antimicrobial peptides are considered a prospective solution to the crisis of antimicrobial resistance. In this study, we evaluated the antimicrobial efficiency of an ApoE mimetic peptide, COG1410, which has been confirmed to exhibit strong neural protective activity and immunomodulatory function. COG1410 showed potent antimicrobial activity against pandrug-resistant *Acinetobacter baumannii*, even eliminating large inocula (10^8^ CFU/ml) within 30 min. LC_99.9_ in PBS and 50% pooled human plasma was 2 μg/ml (1.4 μM) and 8 μg/ml (5.6 μM), respectively. Moreover, COG1410 exhibited biofilm inhibition and eradication activity, excellent stability in human plasma, and a low propensity to induce resistance. Although COG1410 easily entered bacterial cytoplasm and bound to DNA nonspecifically, the major mechanism of COG1410 killing was to disrupt the integrity of cell membrane and lead to leakage of cytoplasmic contents, without causing obvious pores on the cell surface or cell lysis. Additionally, transcriptome analysis showed that treatment with COG1410-enriched genes involved a series of oxidation–reduction processes. DCFH-DA probe detected an increased ROS level in the presence of COG1410, indicating ROS was another hit of this AMP. Furthermore, the action of COG1410 did not depend on the electronic interaction with the LPS layer, in contrast to polymyxin B. The strong synergistic interaction between COG1410 and polymyxin B dramatically reduced the working concentration of COG1410, expanding the safety window of the application. *C. elegans* infection model showed that combined therapy of COG1410 and polymyxin B was capable of significantly rescuing the infected nematodes. Taken together, our study demonstrates that COG1410 is a promising drug candidate in the battle against pandrug-resistant *A. baumannii*.

## Introduction

*Acinetobacter baumannii* is a Gram-negative opportunistic pathogen, causing serious nosocomial infections among immunocompromised patients (Alsan and Klompas, 2010). A striking characteristic of this bacterium is its capability to develop antimicrobial resistance rapidly and survive on the dry surfaces of medical facilities for weeks. As early as 2009, carbapenem-resistant *A. baumannii* was listed among the six high-threat pathogens “ESKAPE” by the Infectious Diseases Society of America, calling for global cooperation in response (Boucher et al., 2009). Particularly, the emergence of chromosomally encoded and plasmid-mediated polymyxin resistance in *A. baumannii* breaks through the last resort of defense (Lima et al., 2018). Additionally, *A. baumannii* possesses a strong capability to form biofilm on either biotic or abiotic surfaces, encasing bacteria in the extracellular matrix, which makes treatment options more difficult (Colquhoun and Rather, 2020; Upmanyu et al., 2022). At present, the prevalence of *A. baumannii* infection is considered a major and complex issue of global health (Perez et al., 2007; Hernández-González and Castillo-Ramírez, 2020). There is an urgent and immediate need for new antimicrobials with activity against this pandrug-resistant (PDR) pathogen.

However, the current pipeline of novel antibiotic development is stagnant, which could not keep pace with these evolving pathogens. The pharmaceutical field turns to seek non-conventional anti-infective agents. The antimicrobial peptides (AMPs) have drawn a lot of attention as novel chemotherapeutic candidates because of their unique mechanisms of action (Mahlapuu et al., 2016). It is known that the sources of AMPs are diverse, ranging from prokaryotes to humans (Zhang and Gallo, 2016). One of the most promising AMPs is the protein mimetic peptide, which originates from a functional protein but shows bioactive activity similar to the intact protein. After chemical modifications, these mimetic peptides have increased proteolytic stability, biological utility, and low cytotoxicity (Groß et al., 2016). Apolipoprotein E (ApoE) is a well-known glycosylated protein in humans that serves as a major lipid transporter in the circulatory system and as an immune modulator in the central nervous system (Liu et al., 2013). After traumatic brain injury (TBI), ApoE regulated the microglial activation and inflammatory response by responding to a variety of acute or chronic injuries in the brain (Tensaouti et al., 2020). Further studies showed that the proteolytic fragments or synthetic peptides derived from the full length of ApoE exerted neuroprotective, immunomodulatory, and antimicrobial activities (Kelly et al., 2007; Laskowitz et al., 2007; Wang et al., 2013; Pane et al., 2016). In particular, the fragment containing the receptor-binding domain of ApoE (130–162) was found to have direct antibacterial activity (Azuma et al., 2000). ApoE_130−162_ was found to be active against *Escherichia coli* but to a lesser extent against *Pseudomonas aeruginosa* (Azuma et al., 2000). Since then, different peptides with different combinations and truncations based on ApoE were expressed or synthesized, with or without modification. Their antimicrobial activities are summarized in Supplementary Table S1. Remarkably, only ApoE_23_ showed potent activity against *A. baumannii* with an MIC of 2 μM.

COG1410 is one of the ApoE-based synthetic peptides, located between residues 138–149 of the ApoE N-terminal domain with aminoisobutyric acid (Aib) substitutions at positions 140 and 145 (Laskowitz et al., 2007). This peptide is originally modified based on COG133 (133–149 of ApoE) to extend the therapeutic window of post-TBI (Laskowitz et al., 2007), which has demonstrated neuroprotective activity in several models of brain injuries, including intracerebral hemorrhage and focal brain ischemia (Laskowitz et al., 2007, 2012; Tukhovskaya et al., 2009). By reducing inflammation and apoptosis, COG1410 enhanced retinal ganglion cell survival and alleviates early brain injury (Wu et al., 2016; Kuai et al., 2019). Furthermore, COG1410 was shown to possess the ability to target the blood–brain barrier (BBB) (Zhang et al., 2020). Recently, it was fused with the Aβ-binding region to form a multi-strategy peptide, which enhanced the BBB targeting efficiency and ameliorated neurologic damage in the Alzheimer's disease model of mice (Zhang et al., 2021). Therefore, COG1410 has been considered a promising therapeutic agent for diseases related to neural injury. However, whether COG1410 has an antibacterial activity like other ApoE analogs remains unknown. In this study, we aim to investigate its antibacterial efficacies and mechanisms of action against PDR *A. baumannii*.

## Materials and methods

### Strains and cultures

The strains evaluated in this study are listed in Table 1. Strain stocks were maintained at −80°C in 10% glycerol. Bacteria were streaked on fresh plates before each experiment. Most of them were cultured in LB broth containing 10 g/L NaCl at 37°C, except for *Mycobacteria* in 7H9 broth and *Enterococcus* in BHI broth. *Porphyromonas gingivalis* was cultured in BHI broth supplemented with 1 μg/ml vitamin k1, 5 μg/ml hemin, and 5 mg/ml L-cysteine hydrochloride at 37°C in the anaerobic chamber. The minimal inhibitory concentrations (MICs) of antimicrobials were determined using the microdilution method in the corresponding broth.

**Table 1:** The bactericidal spectrum of COG1410.

**Gram type**	**Strains**	**MIC (μg/ml)**
G+	MDR S*treptococcus pneumoniae* 10–58	>256
G+	methicillin-resistant *Staphylococcus aureus* 14–55	>256
G+	*Staphylococcus aureus* ATCC29213	>256
G+	vancomycin-resistant *Enterococcus faecalis* 11–34	32
G+	vancomycin-resistant *Enterococcus faecium* 11–47	16
G+	*Mycobacterium tuberculosis* H37Rv	16
G+	MDR *Mycobacterium tuberculosis* 6–45	16
G+	*Mycobacterium smegmatis* MC^2^155	16
G+	*Bacillus subtilis* 168	1
G–	PDR *Klebsiella pneumoniae* 84	>256
G–	EBSL-producing *Klebsiella pneumoniae* YQ7	>256
G–	MDR *Klebsiella oxytoca* 7–85	>256
G–	MDR *Serratia mucosa* YQ15	>256
G–	EBSL-producing *Proteusbacillus vulgaris* YQ19	>256
G–	MDR *Pseudomonas aeruginosa* 14–61	>256
G–	*Pseudomonas aeruginosa* PAO1	>256
G–	*Porphyromonas gingivalis* ATCC33277	32
G–	MDR *Enterobacter cloacae* 7–86	64
G–	*Escherichia coli* ATCC25922	64
G–	MDR *Citrobacter freundii* 7–83	16
G–	*Acinetobacter baumannii* ATCC19606	16
G–	PDR *Acinetobacter baumannii* YQ4	16

*MDR, multidrug resistant; PDR, pandrug resistant; EBSL, extended-spectrum β-lactamase*.

The pandrug-resistant *A. baumannii* YQ4 was collected from the clinical laboratory of the second hospital in Nanjing. The complete genome was sequenced by Nanopore, and the data were deposited in the GenBank with accession number CP053033.

### Peptide synthesis, purification, and identification

The COG1410 peptide was synthesized with conventional solid-phase peptide synthesis at a purity of 95% and qualified by HPLC and mass spectrometry in Polypeptide Labs (San Diego, CA). COG1410 is acetyl-AS-Aib-LRKLAib-KRLL-amide, which is derived from apoE residues 138–149 with Aib (amino-isobutyric acid) substitutions at positions 140 and 145. For all experiments, peptides were dissolved in sterile saline immediately before use.

### Antibacterial activity of COG1410

For dynamic time-kill assay, the overnight culture of *A. baumannii* YQ4 was transferred to fresh broth with an initial OD_600_ of 0.01 and grown to the logarithmic phase. About 1 ml of culture was harvested, washed two times with 1 × phosphate-buffered saline (PBS, pH 7.4, containing 8 mM NaH_2_PO_4_, 2 mM KH_2_PO_4_, 2.6 mM KCl, and 136 mM NaCl), and finally suspended in 1 ml PBS with OD_600_ of 0.5, which approximately corresponds to 1 × 10^8^ CFU/ml. The suspension was supplemented with 16 μg/ml (1 × MIC) or 80 μg/ml (5 × MIC) of COG1410, respectively, and incubated at 37°C without shaking. Aliquots of 100 μl were, respectively, taken at 0, 5, 10, and 30 min, serially diluted in PBS, and plated on LB agar. The CFU was counted after incubation at 37°C for 18 h. A concentration of 16 μg/ml (1 × MIC) or 80 μg/ml (5 × MIC) of polymyxin B was used as a positive control. Three independent experiments were performed.

The antibacterial activity of COG1410 in plasma was performed as described previously (De Breij et al., 2018). Briefly, the log-phase culture of *A. baumannii* YQ4 was exposed to different concentrations of COG1410 in PBS or PBS supplemented with 50% (v/v) pooled human plasma. After incubation with shaking at 200 rpm at 37°C for 2 h, the CFU was counted on LB agar. LC_99.9_ indicates the lowest peptide concentration that kills ≥99.9% of bacteria. The experiments were conducted independently in triplicate.

### Biofilm inhibition and eradication assay

Static biofilm inhibition was performed as described previously (Gaddy and Actis, 2009). Briefly, the log-phase culture of *A. baumannii* YQ4 was prepared as described above. About 200 μl of culture was seeded in each cell of the 96-well PVC plate with OD_600_ of 0.01 (ca. 1 × 10^6^ CFU/ml), which was exposed to different COG1410 solutions with the final concentrations ranging from 0.5 to 128 μg/ml. Each concentration was determined in eight wells. After incubation at 37°C for 48 h, the planktonic bacteria were removed by washing three times with sterilized water, followed by fixing in methanol for 15 min and staining with 0.1% crystal violet (CV) for 15 min. Excess CV was washed off, and the bound CV was eluted from the biofilm into 150 μl of 95% ethanol and quantitated spectrophotometrically at 600 nm using a Biotek Synergy H1 plate reader. The experiments were performed in triplicate.

Biofilm eradication assay was performed as described previously (Mwangi et al., 2019). *A. baumannii* YQ4 culture of log phase was diluted in fresh LB broth to OD_600_ 0.01 and dispensed to a 96-well PVC plate with 200 μl per well. Plates were incubated at 37°C for 48 h and washed with PBS three times. Serial dilutions of COG1410 (0.5–128 μg/ml) were prepared using the same media, and 200 μl were dispensed into each well. Each concentration was determined in eight wells. LB without COG1410 was used as untreated control. After incubation for another 24 h, the remaining biofilm was quantified as described above. The experiments were performed in triplicate.

### Stability of COG1410 in human plasma

In order to determine the stability in human plasma, COG1410 was dissolved in1 ml of 100 % human plasma with the final concentration of 10 mg/ml and incubated at 37°C without shaking. Aliquots of 100 μl were taken at 0, 1, 2, 4, 6, 8, and 10 h. The log-phase culture of *A. baumannii* YQ4 was prepared as described above. About 200 μl of culture was mixed with 6 ml of 0.8% soft agar to make a two-layered plate. After air drying for 30 min, four 6-mm paper disks were placed on the top, and 6-μl aliquots of COG1410 at different time points were dropped on the paper disks. After 18 h of incubation at 37°C, the inhibition zones were recorded by a digital camera, and the inhibition diameters were measured by image J. Three independent experiments were performed.

### Scanning electron microscopy (SEM) and transmission electron microscopy (TEM)

The log-phase culture of *A. baumannii* YQ4 was prepared as described above, harvested, and washed once with PBS. The pellets were suspended in PBS supplemented with 1 × COG1410 (16 μg/ml) or 1 × polymyxin B (16 μg/ml) and incubated at 37°C for 30 min. The untreated culture was used as a positive control to observe the morphology of the intact cell. Then, the pellets were fixed with 4% paraformaldehyde and 2.5% glutaraldehyde in 0.1 M sodium cacodylate and stored at 4°C overnight. After washing three times with PBS, the cells were post-fixed in 1% osmium at 4 °C for 1 h. After washing in distilled water for 1 h, the bacteria were dehydrated in the ascending concentrations of ethanol. Finally, these samples were transferred to ThermoFisher Quattro SEM for image collection after tert-butanol freeze-drying and sputter coating.

The bacteria were treated with COG1410 or polymyxin B as above. Samples were then fixed in 4% paraformaldehyde and 2.5% glutaraldehyde in 0.2 M sodium cacodylate buffer (pH 7.2), post-fixed in 1% buffered osmium tetroxide, dehydrated in gradient ethanol, embedded in epoxy resin, and polymerized at 60°C for 2 days. Ultrathin sections (50–70 nm) of samples were made and placed on the copper grids, stained with uranyl acetate and lead citrate, and transferred to ThermoFisher Talos L120C TEM for image collection.

### Confocal laser scanning microscopy

The damage to the cytoplasmic membrane was determined as described previously (Farkas et al., 2017), with the Live/Dead BacLight bacterial viability kit (Invitrogen L7012). The log-phase culture of *A. baumannii* YQ4 was washed two times and suspended in PBS with the final OD_600_ of 0.1. The suspension was incubated with 1× MIC COG1410 (16 μg/ml) at 37°C for 30 min. In addition, 5× MIC polymyxin B (80 μg/ml) and untreated cells were used as positive control and negative control, respectively. Then, the cells were stained with 7.5 μM SYTO-9 and 30 μM propidium iodide (PI) in dark for 15 min. In total, 5 μl of culture was spotted on a clean slide coated with a thin layer of 1% agarose. The fluorescence was observed by Olympus FV3000 confocal laser scanning microscope. For SYTO-9, the excitation and emission were 483 and 503 nm, respectively. For PI, the excitation and emission were 493 and 636 nm, respectively.

To explore the localization of COG1410 in bacteria, the log-phase cultures of *A. baumannii* YQ4, *K. pneumonia* ATCC2146, *S. aureus* ATCC29213, and *E. faecium* 11–47 were prepared, washed, and suspended in PBS with a final OD_600_ of 0.1. These cultures were exposed to FITC-labeled COG1410 (8 μg/ml) for 30 min at room temperature, followed by co-staining with 1.6 μM membrane dye FM4-64. For each strain, 5 μl of culture was spotted on a clean slide coated with a thin layer of 1% agarose. The fluorescence was observed by Olympus FV3000 confocal laser scanning microscope. For FITC, the excitation and emission were 488 nm and 525 nm, respectively. For FM4-64, the excitation and emission were 558 nm and 734 nm, respectively.

### ATP leak assay

ATP leak assay was performed using the Enhanced ATP Assay Kit of Beyotime (S0027) according to the manufacturer's manual. Briefly, the log-phase culture of *A. baumannii* YQ4 was prepared, washed, and suspended in PBS as above. This suspension was exposed to 16 μg/ml of COG1410, 16 μg/ml of polymyxin B, and 32 μg/ml of tigecycline, respectively, and incubated at 37°C for 30 min. The supernatant was harvested and used for the measurement of ATP levels. In total, 100 μl of supernatant was mixed with 100 μl of working solution, and the chemiluminescence was measured by using a Biotek Synergy H1 plate reader. The untreated sample was set as a negative control. The experiments were performed in triplicate.

### DNA-binding assay

Gel retardation experiments were conducted as previously described (Yan et al., 2013). Briefly, 300 ng of the plasmid pUC18 was mixed with different concentrations of COG1410 in 30 μl of buffer (10 mM Tris-HCl and 1 mM EDTA buffer, pH 8.0) and incubated at room temperature for 30 min. The reaction mixtures were mixed with 1 × native loading buffer and subjected to 1.5% agarose gel electrophoresis. The migration of DNA was detected by the fluorescence of GelRed dye.

### RNA-seq analysis

To determine the effect of COG1410 on gene transcription, *A. baumannii* YQ4 was cultured in 50 ml of M9 medium supplemented with 20% glucose as the sole carbon source, treated with or without 1/4 MIC of COG1410 (4 μg/ml). The experiment was conducted in triplicate. When the OD600 reached 0.8, the cells were collected by centrifugation at 4°C and frozen in liquid nitrogen. The samples were shipped with dry ice to Guangdong Magigene Biotechnology Co., Ltd. (Guangzhou, China). Total RNA was extracted and purified using the TransZol Up Plus RNA Kit and EasyPure RNA Purification Kit, and rRNA was removed using Ribo-Zero rRNA Removal Kit, according to the manufacturer's instructions. The whole libraries for Illumina were generated by using NEB Next ^®^ Ultra™ Directional RNA Library Prep Kit. After cluster generation, the library was sequenced on an Illumina Novaseq6000 platform, and 150 bp paired-end reads were generated. The raw data were filtered by fastp, and rRNA sequences were removed (Chen et al., 2018). Differentially expressed genes were performed using the edgeR. Genes with the FDR 0.05 and |log_2_(fold change)| > 1 were taken as candidate genes. Gene ontology (GO) and Kyoto Encyclopedia of Genes and Genomes (KEGG) enrichment analysis of differentially expressed genes were implemented by the cluster Profiler. The raw data have been deposited to the SRA database with the accession number PRJNA833738.

### ROS detection

The intracellular ROS level was determined by the Reactive Oxygen Species Assay Kit of Beyotime Biotechnology (S0033S), according to the manufacturer's instructions. The log-phase culture of *A. baumannii* YQ4 was harvested and washed in PBS, and then diluted 10 times, approximately 10^7^ CFU/ml. In total, 1 μl of DCFH-DA (10 μM) was added to 1 ml of cell culture and incubated at 37°C for 20 min. Then the fluorescence probe was fully removed by washing with PBS three times and resuspending in PBS, followed by the addition of COG1410 (16 μg/ml) or water. Rosup (50 μg/ml) was positive control in the kit. The culture was incubated at 37°C for 30 min. The fluorescence intensity was measured by a plate reader at an excitation wavelength of 488 nm and an emission wavelength of 525 nm. The experiment was performed in duplicate.

### Evaluation of induction of resistance through serial passage

To evaluate the drug resistance barrier of COG1410, *A. baumanii* YQ4 samples were cultured in LB broth with constant shaking at 150 rpm at 37°C, with exposure to sub-MIC COG1410 or polymyxin B. In total, 20 μl of culture was transferred into 2 ml of fresh medium every day. The initial concentration of the tested compounds was set up as 1/32 MIC and doubled after every 10 passages. Then, 1 ml of bacterial culture was stored in 10% sterile glycerol at −80°C after every five passages. The MIC values of collected cultures and original strains were measured through the microdilution method.

### Hemolysis assay and cytotoxicity assay

The human red blood cell (RBC) hemolytic activity of COG1410 was measured according to the protocol described previously with minor modifications (Oddo and Hansen, 2017). The anti-coagulated (citrate) whole blood sample was pelleted by centrifugation at 700 *g* for 8 min, washed three times with PBS, and suspended in 0.5% (vol/vol) PBS. Then, 75 μl of RBC suspension was transferred to each well of the V-bottom 96-well plate, where equal volumes of COG1410 were prepared with 2-fold dilution in PBS. The highest concentration was 512 μg/ml (363 μM). PBS and Triton X-100 (0.1%) were used as negative and positive controls, respectively. The plate was incubated at 37°C for 1 h, followed by centrifugation at 1,000 rpm for 5 min at 4°C. Aliquots of 60 μl of the supernatant from each well were quickly transferred to a new flat-bottom 96-well plate. The optical absorbance at OD_414_ was measured with a microplate reader (BioTek, synergy H1). The hemolysis percentage was then normalized with respect to the averaged negative (0%) and positive (100%) controls. Three independent experiments were conducted.

The cytotoxicity of COG1410 on normal liver cell L02 was assessed by Cell Counting Kit 8 (Solarbio, CA1210) according to the manufacturer's manual. Briefly, 100 μl of human hepatic L02 cells was seeded in the 96-well plate with 4 × 10^3^ per well in RPMI-1640 medium containing 20% FBS and incubated at 37°C in a 5% CO_2_ atmosphere for 24 h. The cells without exposure to peptides were used as a negative control. Then, the L02 cells were incubated with different concentrations of COG1410 for another 24 h. Then, 10 μl of CCK8 solution was added to each well. After 2 h of incubation, the optical absorbance at 450 nm was measured with a BioTek synergy H1 plate reader. The cell activity was expressed as the percentage of mean absorbance by cells exposed to the peptide with respect to the results obtained by incubation with the control. The experiments were done in triplicate.

### Synergy with antimicrobials

The checkerboard method was performed to determine the fractional inhibitory concentration (FIC) index between COG1410 and other antimicrobials. The 2-fold serial dilutions of antimicrobials and COG1410 were conducted and mixed in the 96-well plate. The log-phase culture of *A. baumannii* YQ4 was added to each well with an initial OD_600_ of 0.01. The plate was incubated at 37°C for 20 h, and the FIC values were calculated based on the equation: FIC = MIC (COG1410 in combination)/MIC (COG1410 alone) + MIC (antimicrobial in combination)/MIC (antimicrobial alone). The experiments were conducted twice independently.

### Nematode killing assay

The wild-type strain of *Caenorhabditis elegans* (Bristol N2) was used in this assay. The relative experimental manipulation was done according to the previously described protocols (Stiernagle, 2006). The cultures of *C. elegans* were grown on a nematode growth medium (NGM) with *E. coli* OP50 lawn as a food source at 20°C. For synchronization, *C. elegans* eggs were harvested and hatched to stage L1 in M9 medium at 20°C, then transferred to *E. coli* lawns to grow to stage L4. For the *in vivo* killing assay, the synchronized L4 nematodes were harvested from a few NGM plates and transferred to 15 ml of M9 medium containing 20% LB, 1 × 10^9^ log-phase cells of *A. baumannii* YQ4, and 10 μM FeCl_3_. The infection model was established at 20°C for 24 h. The culture of *E. coli* OP50 was used as a negative control. The pre-infected nematodes were washed two times with M9 medium and dispensed on a 60-mm NGM agar plate with 30 nematodes each, where different concentrations of COG1410 and/or polymyxin B, as well as 2 mM 5-Fluoro-2′-deoxyuridine-floxuridine (FUDR), were supplemented. The uninfected nematodes were used as a positive control to determine the worm's lifespan. Live and dead or missing nematodes were counted and recorded through a stereomicroscope every 24 h for 16 days. The survival curve of *C. elegans* was analyzed by Kaplan–Meier using GraphPad Prism 9. The *in vivo* killing assays were performed in triplicate.

## Results

### COG1410 possesses a broad spectrum of antimicrobial activity

COG1410 is a synthetic cationic peptide with a simple alpha helix and is composed of 12 amino acids, acetyl-AS-Aib-LRKL-Aib-KRLL-amide, including 4 positively charged, 5 nonpolar, and 1 polar amino acids, as well as two unnatural amino acids, Aib. The antimicrobial activity of COG1410 was determined by measuring the minimal inhibition concentration (MIC) values on a panel of Gram-positive and Gram-negative strains (Table 1). In the case of Gram-positive strains, COG1410 inhibited the growth of *Bacillus subtilis*, vancomycin-resistant *Enterococcus faecalis* and *Enterococcus faecium*, and *Mycobacterium smegmatis* with MICs ranging from 1 to 32 μg/ml. As for Gram-negative bacteria, COG1410 showed antimicrobial activity against *Enterobacter cloacae, Escherichia coli, Citrobacter freundii*, and even anaerobe, *Porphyromonas gingivalis*, with MICs ranging from 16 to 64 μg/ml. In contrast, COG1410 was inactive against *Streptococcus pneumoniae*, methicillin-sensitive *Staphylococcus aureus*, methicillin-resistant *S. aureus* (MRSA), *Klebsiella pneumoniae*, and *Pseudomonas aeruginosa*. Remarkably, COG1410 could kill the pandrug-resistant (PDR) *Acinetobacter baumannii* YQ4, with MIC and minimal bactericidal concentration (MBC) of 16 μg/ml (11.3 μM). We continued to test the other 107 clinically collected *A. baumannii* strains and found that the MICs of COG1410 ranged from 16 to 32 μg/ml (Supplementary Table S2).

### COG1410 shows potent and quick bactericidal efficacy against PDR *A. baumannii in vitro*

**Figure 1:**
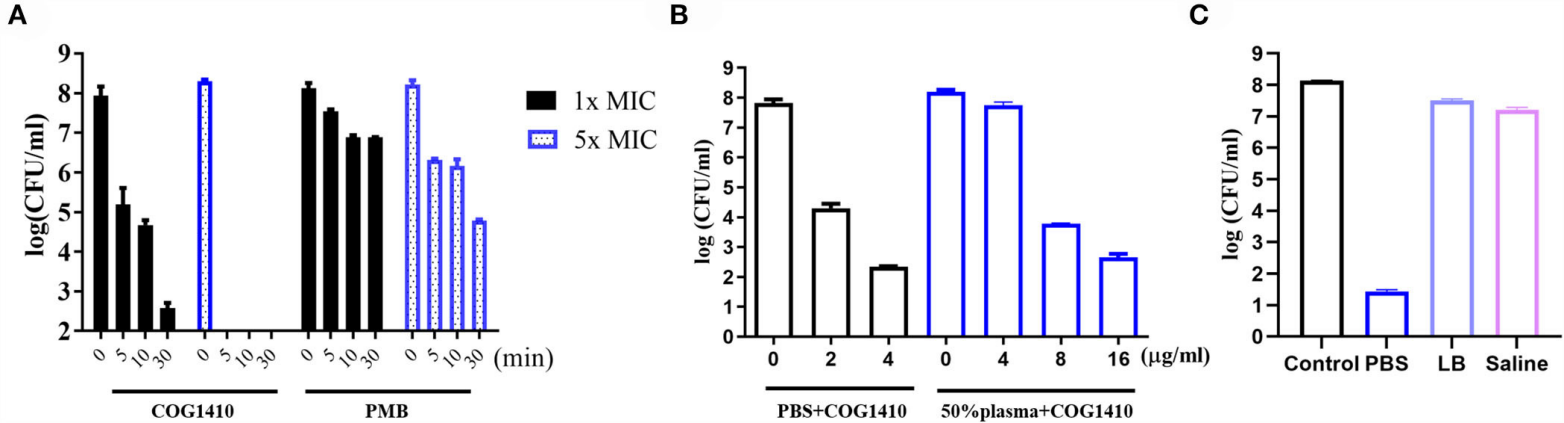
Antimicrobial activity of COG1410. **(A)** *In vitro* killing kinetics of COG1410 and polymyxin B against PDR *A. baumannii* YQ4 in PBS at 1× MIC and 5 × MIC, respectively. **(B)** Bactericidal efficacy of COG1410 in PBS with or without 50% human plasma. The CFU/ml was calculated after incubation at 37°C for 2 h. **(C)** Bactericidal efficacy of COG1410 in different conditions was determined. PMB, polymyxin B. Each experiment was done in triplicate, and the values are represented as mean ± SD.

The *in vitro* time-kill kinetics of COG1410 against the PDR *A. baumannii* YQ4 were analyzed. The 1× MIC of COG1410 decreased by almost 3 log CFU/ml within 5 min in 20 μM phosphate-buffered saline (PBS) (Figure 1A). When the concentration of AMP increased to 5 × MIC, the inoculum (1 × 10^8^ CFU/ml) was completely eliminated within 5 min. Within 30 min, 1× MIC of COG1410 killed 6 log CFU/ml. In comparison, 1 × and 5× MIC of polymyxin B decreased 2 log CFU/ml and 4 log CFU/ml after 30 min, respectively (Figure 1A). These data showed that COG1410 acted more rapidly than polymyxin B.

To determine the efficacy of COG1410 in the pooled human plasma, we measured its LC_99.9_ value (killing 99.9% within 2 h of incubation) in PBS with or without 50% human plasma. As shown in Figure 1B, 2 μg/ml (1.4 μM) concentration of COG1410 achieved 99.9% killing in PBS. Consistently, the antimicrobial activity of this AMP was reduced in the presence of human plasma, with LC_99.9_ up to 8 μg/ml (5.6 μM).

To address the activity of COG1410 in other conditions, we determined the bactericidal efficacy of COG1410 in PBS, LB broth, and saline, respectively. Compared to almost complete elimination in PBS, 1× MIC of COG1410 only killed 78.6% and 78.3% of the *A. baumannii* population in LB broth (250 mM NaCl) and saline (225 mM NaCl) after incubation for 2 h, respectively (Figure 1C). These data suggested that high salt concentrations might affect the bactericidal effect of COG1410.

### COG1410 maintains stability in human plasma

In order to determine the stability of COG1410 in human plasma, a 10 mg/ml concentration of COG1410 was incubated with 100% human plasma at 37°C. Samples were retrieved at different time points and dropped on paper disks. The antimicrobial activity was evaluated by measuring the diameter size of the inhibition zone. To our interest, the inhibition diameters of COG1410 did not significantly change within the first 2 h (Supplementary Figure S1). Until 4 h, the activity reduced by 6.8%. After 10 h of incubation, 20% of activity was lost. These data suggested that COG1410 was very stable in human plasma.

### COG1410 exhibits biofilm inhibition and eradication activity

**Figure 2:**
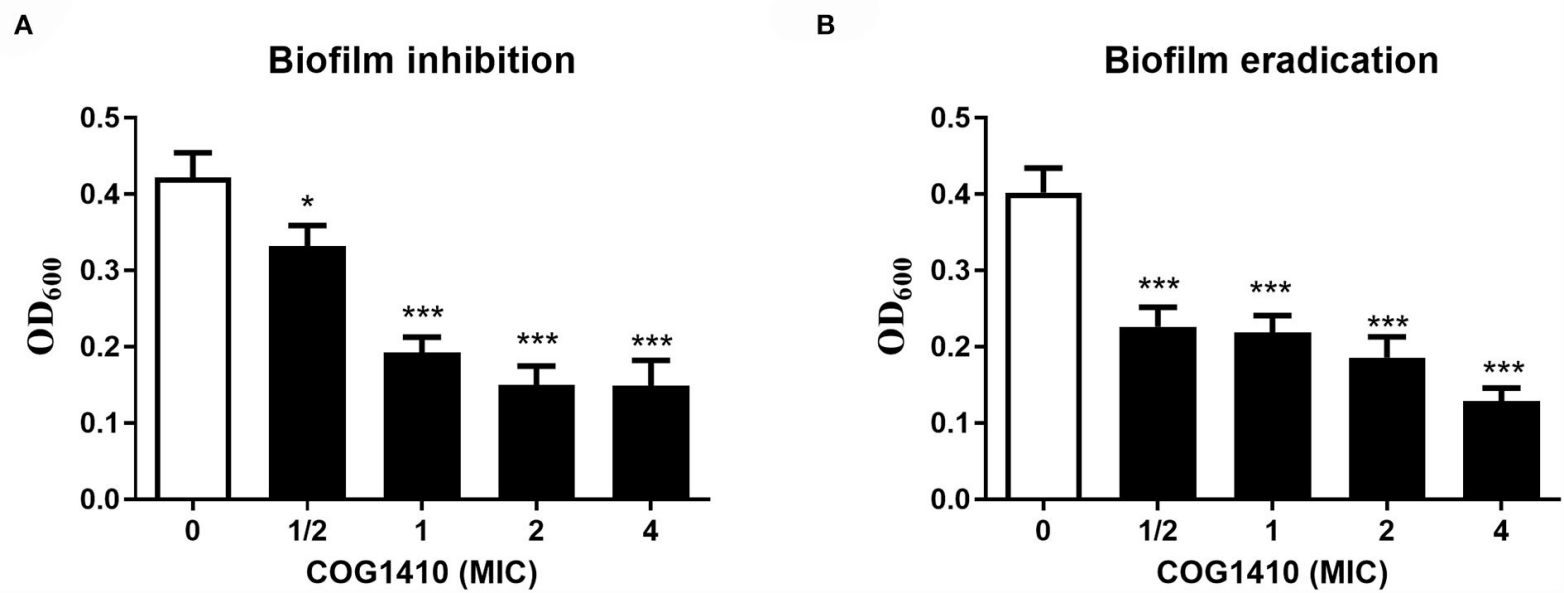
COG1410 exhibited biofilm inhibition and eradication activities against PDR *A. baumannii*. **(A)** Prevention of biofilm formation by COG1410. Results were expressed as the biofilm mass measured using crystal violet staining (OD_600_). **(B)** Eradication of established biofilm. Data were represented mean ± SD of eight replicates from three independent experiments. The statistical significance between each treatment and control was analyzed by Student's *t*-test (unpaired), *p < 0.5, *** *p* < 0.001.

Bacterial biofilm formation is often associated with chronic wound infections and drug resistance (He et al., 2015). To investigate the efficacy of COG1410 against biofilm, we measured the biofilm mass formed by *A. baumannii* YQ4 by crystal violet staining. COG1410 inhibited biofilm formation in a dose-dependent manner (Figure 2A). The 1/2 MIC of COG1410 significantly reduced biofilm formation. The 1× MIC of COG1410 reduced 55% of biofilm mass, compared with the untreated control.

For preformed biofilm, 1× MIC of COG1410 dispersed approximately 46% of the mature biofilm. If the AMP concentration increased to 4 × MIC, more than 88% of biofilm was eradicated (Figure 2B). Taken together, COG1410 was a promising antibiofilm agent.

### COG1410 treatment increases cell membrane permeability

**Figure 3:**
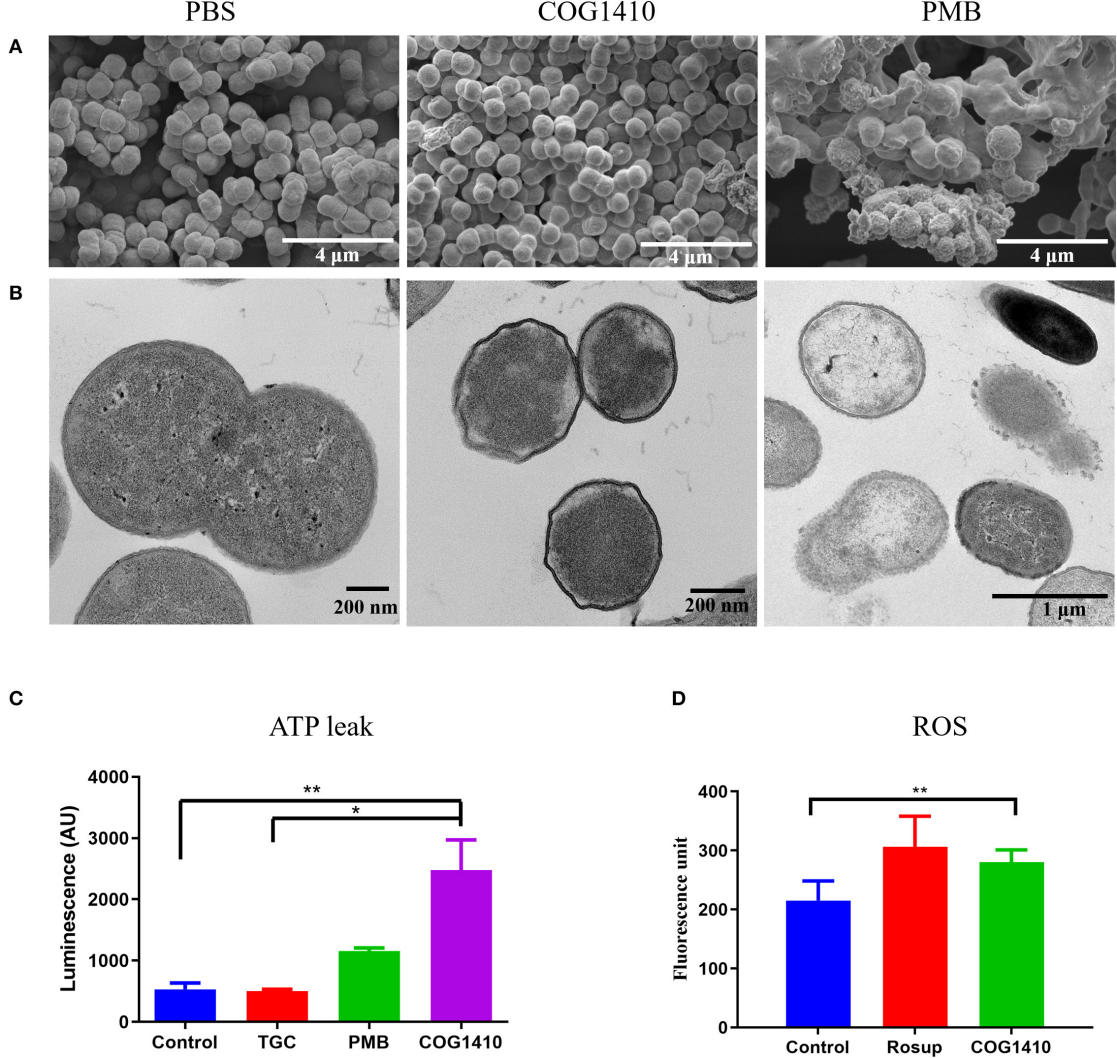
COG1410 treatment permeabilized cell membrane of PDR *A. baumannii*. SEM **(A)** and TEM **(B)** observation of *A. baumannii* YQ4 exposed to 1× MIC COG1410 or 1× MIC polymyxin B. The cells in PBS were negative control. **(C)** The effect of 1× MIC COG1410 on ATP release from *A. baumannii* YQ4. The 1× MIC polymyxin B and 1× MIC tigecycline (TGC, 32 μg/ml) were used as positive and negative controls for ATP leak, respectively. Untreated cells also acted as the negative control. **(D)** Measurement of ROS level by DCFH-DA probe in the presence or absence of COG1410 (16 μg/ml). Rosup is the positive control in the kit. The statistical significance between each treatment and control was analyzed by one-way ANOVA method with multiple comparisons, * *p* < 0.05, ** *p* < 0.01.

To assess the mechanism of action of COG1410, we first examined the cell morphology by scanning electron microscopy (SEM). As illustrated in Figure 3A, untreated *A. baumannii* cells looked like peanuts with small spike-like patterns on the surfaces. As a control, polymyxin B treatment lead to cell lysis and release of cell contents. In contrast, the majority of COG1410 treated cells were intact, with little debris observed. No formation of pores was detected on the cell surfaces. These data indicated that COG1410 might not cause pore formation or cell lysis.

In order to determine the integrity of the cell membrane, we observed the same batch of treated cells by transmission electron microscopy (TEM). The untreated cells had intact cytoplasm membranes. Exposure to polymyxin B gradually caused the collapse of cell walls in most of the cells (Figure 3B). However, the COG1410-treated cells became wrinkled and smaller compared with untreated cells. Additionally, it seemed that the inner membrane was separated from the cell wall.

To verify the permeabilization of the plasma membrane by COG1410, we decided to stain COG1410-treated *A. baumannii* YQ4 cells with two fluorescent nucleic acid dyes, SYTO-9 and propidium iodide (PI). The former could stain both live and dead cells and show green fluorescence, but the latter only enters non-living cells and emits red fluorescence. As expected, untreated cells showed green fluorescence, indicating all of them were living. Both COG1410- and polymyxin B-treated cells showed red fluorescence, which suggested that the cells were dead (Supplementary Figure S2). It is worthy to note that no green fluorescence was observed in these treated dead cells after staining with SYTO-9, which might be due to competition of PI. Thus, this staining assay confirmed that COG1410 permeabilized the bacterial membrane.

Membrane disruption can be further characterized by measuring the amount of the intracellular components leaked out of the bacterial cells. To address the mechanism of action of COG1410, we measured the extracellular ATP concentration of cells exposed to 1× MIC of COG1410 by Enhanced ATP Assay Kit (Beyotime). Tigecycline binds to the bacterial 30S ribosome, blocking the entry of transfer RNA, which was used as a negative control (Greer, 2006). As expected, tigecycline-treated cells were similar to untreated cells with regard to the ATP leak. Cells exposed to polymyxin B released more ATP compared to untreated cells. Remarkably, COG1410 treatment lead to ATP leak, which was significantly more than untreated control and tigecycline treatment group (Figure 3C). Taken together, the cationic AMP COG1410 disrupted bacterial cell membrane.

### COG1410 is localized in the cytoplasm

**Figure 4:**
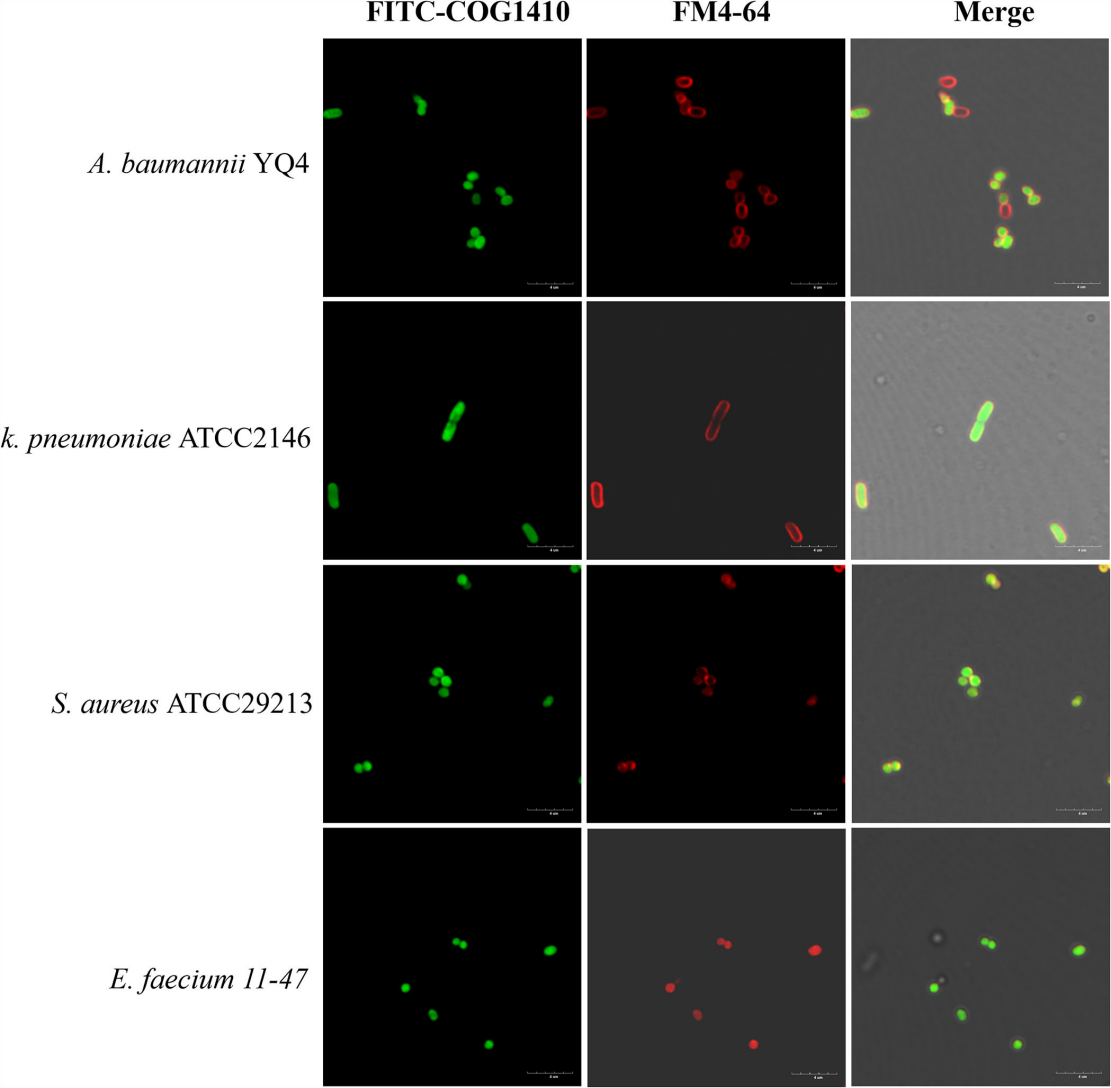
FITC-COG1410 entered the cytoplasm of *A. baumannii, E. faecium, K. pneumoniae*, and *S. aureus*. The bacteria were treated with FITC-COG1410 and stained with FM4-64 dye and observed by CLSM. The green fluorescence indicates the localization of FITC-COG1410 in the cells. Red fluorescence indicated the cytoplasmic membrane. Scale bar: 4 μm.

Besides direct membrane disruption, some sub-MIC AMPs may have cytoplasmic targets (Vasilchenko and Rogozhin, 2019). To address if COG1410 directly binds with bacterial membrane or enters the cytosol, we treated a few bacterial pathogens with an 8 μg/ml concentration of COG1410 for 30 min and then co-stained with red fluorescent membrane dye. COG1410 not only entered the cytoplasm of *A. baumannii* and *E. faecium* but also entered the cells of *K. pneumoniae* and *S. aureus* (Figure 4). COG1410 was active against the former two strains but void against the latter two strains. These findings suggested that COG1410 may bind with the cell membrane of *A. baumannii* more easily than other bacteria or that COG1410 specifically inhibited a cytoplasmic target in *A. baumannii*.

### COG1410 nonspecifically binds with DNA

To address whether the cationic AMP binds with DNA, we performed a gel retardation experiment. The electrophoretic mobility of plasmid pUC18 was checked after incubation with different concentrations of COG1410. Without AMP, the plasmid normally migrated into the gel. In the presence of 1× MIC of COG1410, some amount of DNA remained in the loading well and only part of them migrated. At higher concentrations of COG1410, DNA mobility was completely retarded (Supplementary Figure S3). Taken together, COG1410 was able to bind DNA nonspecifically in a concentration-dependent manner.

### Sub-MIC of COG1410 treatment induces expression of genes involved in the oxidation–reduction process

**Figure 5:**
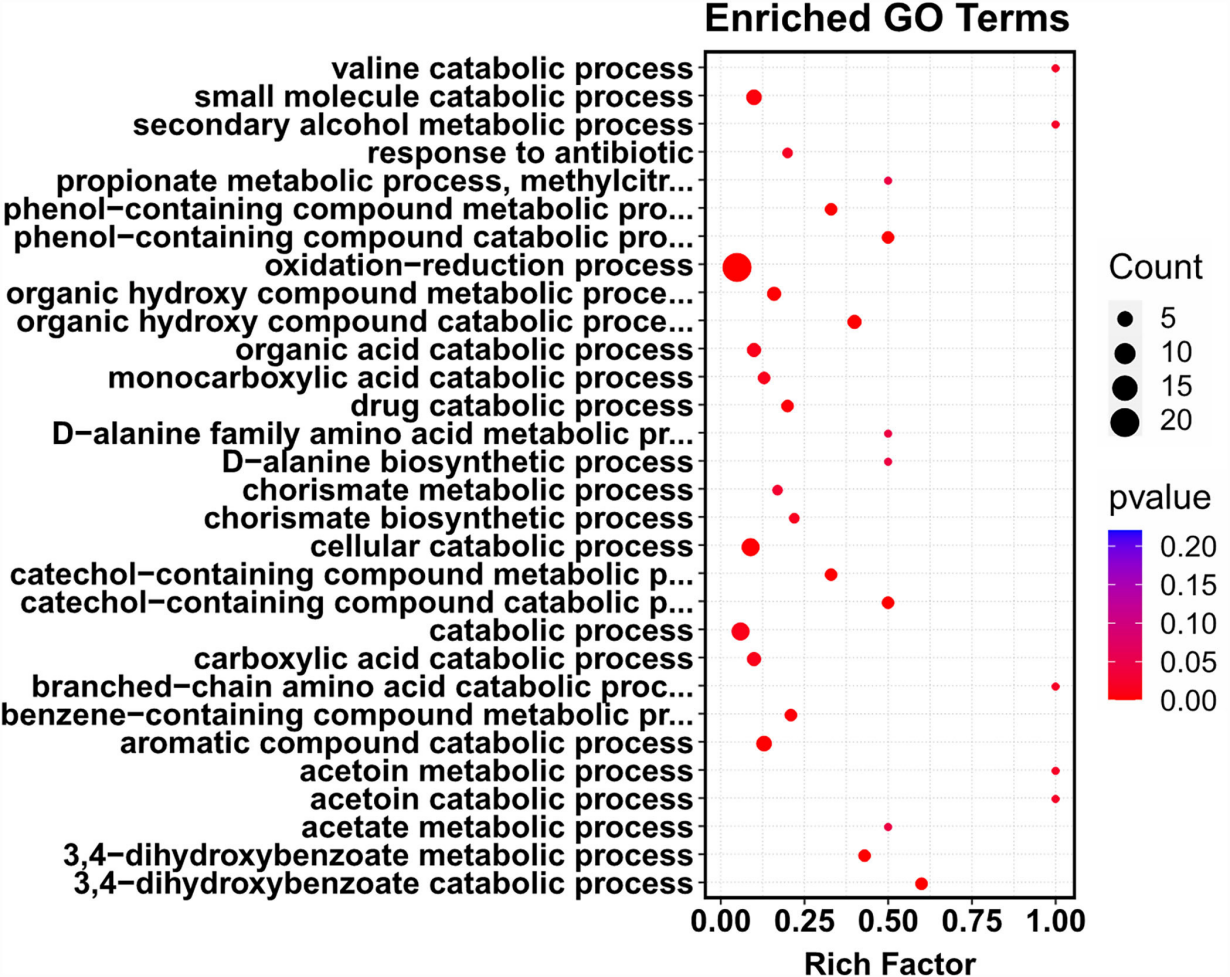
Treatment of COG1410-enriched genes involved in oxidation–reduction process. The whole transcriptome analysis of *A. baumannii* treated or untreated with COG1410 was performed by RNA-seq. The differentially expressed genes (DEGs) were analyzed by using the edgeR (v3.16.5). Gene ontology (GO) enrichment analysis of DEGs was implemented by clusterProfiler (v3.4.4). GO terms with FDR ≤ 0.05 were considered significantly enriched by DEGs.

To further identify the putative intracellular targets of COG1410, RNA-seq was used to compare the transcriptome of *A. baumanii* YQ4 in the presence or absence of 1/4 MIC of COG1410 (4 μg/ml). To reduce interference from the rich medium, the M9 minimal medium was chosen to prepare bacterial culture. The analysis of RNA-seq data identified 92 significantly differentially expressed genes (DEGs) with at least a 2-fold change (Supplementary Table S3). Compared with the untreated control, the transcription level of 55 and 37 genes increased and decreased in the presence of AMP, respectively. The 92 DEGs were classified into 12 categories at GO level 2, such as catalytic activity, cellular anatomical entity, metabolic process, and response to stimulus (Supplementary Figure S4). To our interest, the genes involved in the oxidation–reduction process were enriched (Figure 5). This raised the question of whether the treatment of COG1410 increased reactive oxygen species. To address this concern, we measured the intracellular ROS level using a DCFH-DA probe. As shown in Figure 3D, treatment of COG1410 significantly lead to more ROS production, which indicated that ROS might be another killing mechanism of COG1410.

### COG1410 is highly refractory to induce resistance

For a new antimicrobial compound, its drug resistance barrier is a pivotal parameter, since the bacterial population always tries to develop resistance to survive. Particularly, *A. baumannii* is naturally competent to adsorb exogenous DNA to acquire resistance against many kinds of antibiotics (Domingues et al., 2019). To evaluate the rate of resistance development of *A. baumannii* to COG1410, the PDR strain, YQ4, was serially passaged in the presence of sub-MIC COG1410. Polymyxin B was included as an antibiotic control. As shown in Supplementary Figure S5, the MIC value of polymyxin B increased 64-fold. In contrast, the MIC of COG1410 only increased 4-folds in YQ4 after 55 passages. These data indicated that *A. baumannii* cannot easily gain resistance to COG1410 *via* genetic mutations.

### COG1410 shows low hemolytic activity and medium cell toxicity

**Figure 6:**
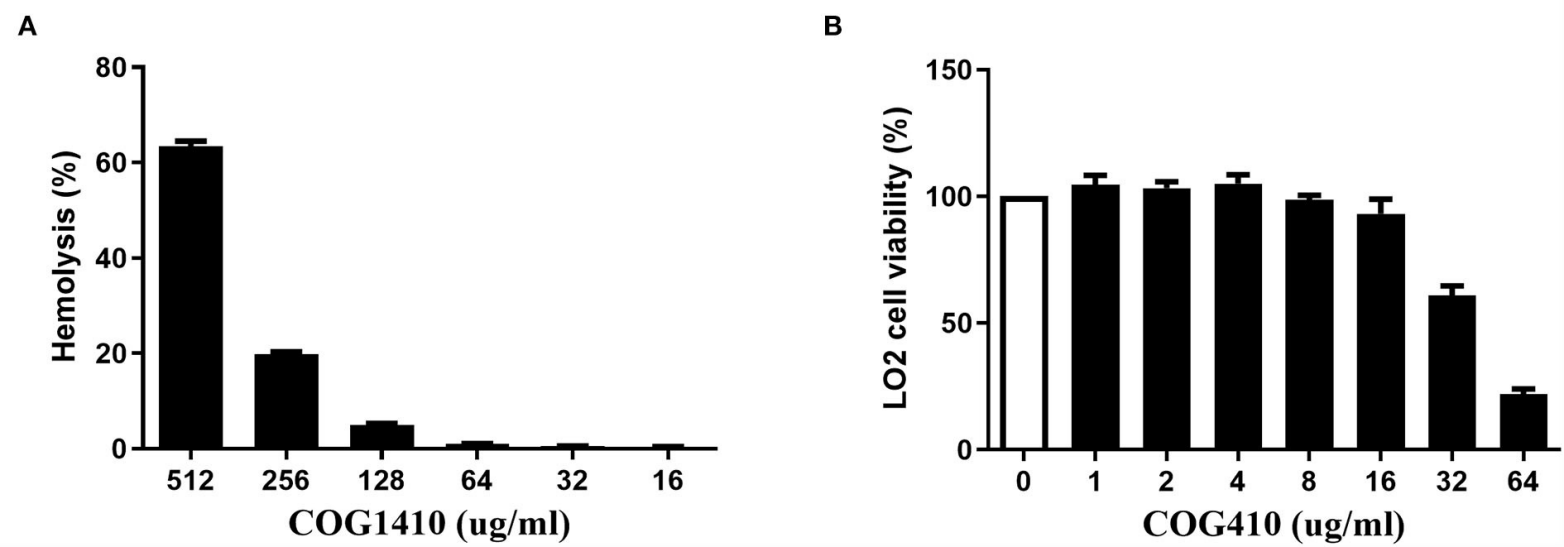
COG1410 exhibited low hemolytic activity and medium cytotoxicity. **(A)** The hemolytic activity was determined by measuring the release of hemoglobin by human erythrocytes at 414 nm, which were exposed to different concentrations of COG1410. PBS and Triton X-100 (0.1%) were used as negative and positive controls, respectively. **(B)** The cytotoxicity of COG1410 was evaluated by measuring the cell viability of normal human hepatic L02 cells treated with the increasing concentration of peptide using the CCK8 assay. Experiments were conducted in triplicate. Data indicated mean ± SD values.

We determined the hemolytic potential of COG1410 by exposing human erythrocytes to different concentrations of COG1410 and measuring the release of hemoglobin. With respect to 100% release of 0.1% Triton X-100 and 0% release of PBS, 128 μg/ml (8 x MIC) only led to <5% hemolysis (Figure 6A). The minimal concentration that caused the lysis of half of the red blood cells (EC_50_) was 441 μg/ml. Therefore, the selectivity index, SI (EC_50_/MIC), was 27.5 for PDR *A. baumannii* YQ4.

The cytotoxic effect of COG1410 on normal human hepatic L02 cells was evaluated by CCK8 assay. As shown in Figure 6B, there was 7% toxicity to the L02 cell at 16 μg/ml. The half-maximal effective concentration (EC_50_) was 58.9 μg/ml.

### The bactericidal action of COG1410 was different from polymyxin B

**Figure 7:**
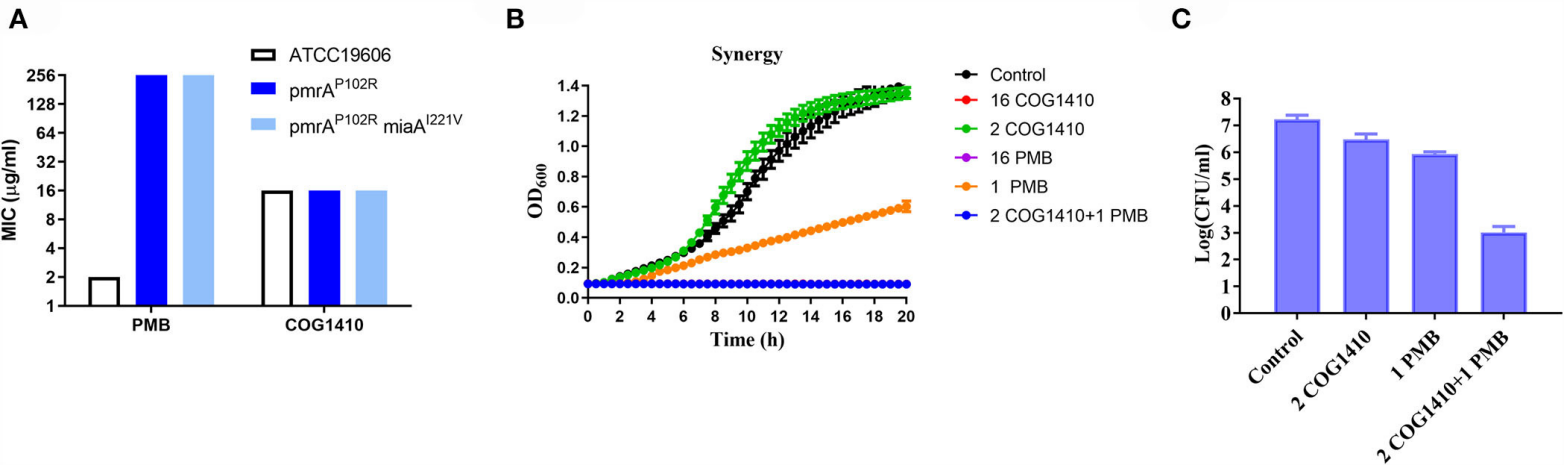
COG1410 exhibited strong synergistic interaction with polymyxin B. **(A)** Modification of LPS did not change the antimicrobial activity of COG1410 against *A. baumannii*. MIC was determined against *A. baumannii* wild-type strain ATCC19606 and the corresponding LPS-defective mutants with *pmrA*^P102R^ and *pmrA*^P102R^*miaA*^I221V^ mutation in LB broth. **(B)** The combination of 2 μg/ml COG1410 and 1 μg/ml polymyxin B could completely inhibit bacterial growth within 20 h in the LB broth. The growth curve was measured in duplicate, with eight wells for each treatment in a 96-well plate each time. The representative one was displayed. **(C)** The combination of COG1410 and polymyxin B significantly reduced the CFU of *A. baumannii* YQ4 in PBS. Experiments were conducted in triplicate. Data indicated mean ± SD values.

To address whether the antimicrobial effect of COG1410 depends on negatively charged lipopolysaccharides (LPSs) on the cell surface, we compared the susceptibility of *A. baumannii* ATCC19606 and two LPS-modified strains, *pmrA*^P102R^ and *pmrA*^P102R^*miaA*^I221V^ (Sun et al., 2020). pmrA mutation led to derepression of PmrC, which encodes lipid A phosphoethanolamine transferase (Arroyo et al., 2011). We found that lipid A modification of LPS did not change the antimicrobial efficacy of COG1410. This was in contrast to polymyxin B, the last resort for MDR Gram-negative bacteria, whose bactericidal effect depends on the electrostatic interaction with LPS (Figure 7A). These data suggest that COG1410 might have a different antibacterial mechanism against polymyxin B and that there is no cross-resistance between them.

### COG1410 shows strong synergy with polymyxin B

To investigate whether COG1410 has a synergistic interaction with conventional antibiotics, we measured the synergy of COG1410 in combination with a few frequently used antibiotics against *A. baumanii* in medical practice. As shown in Table 2, COG1410 displayed synergy with polymyxin B, ceftazidime, and tetracycline with FIC values of 0.13, 0.31, and 0.31, respectively. To our interest, only 2 μg/ml of COG1410 and 1 μg/ml of polymyxin B were able to completely inhibit the growth of the PDR strain (Figure 7B). Time-kill kinetics showed that this combination displayed similar bactericidal activity to 16 μg/ml concentration of COG1410 (Figure 7C).

**Table 2:** Fractional inhibitory concentrations of COG1410 and different antibiotics against the PDR *A. baumannii* strain YQ4.

**Antimicrobial agents**	**MIC**	**FIC with COG1410**
COG1410	16	–
PMB	16	0.13
CIP	256	0.8
CAZ	512	0.31
MERO	32	0.56
TGC	32	1.06
TET	1,024	0.31
AMI	>1,024	>1
GM	>1,024	>1

*PMB, polymycin B; CIP, ciprofloxacin; CAZ, ceftazidime; MERO, meropenem; TGC, tigecycline; TET, tetracycline; AMI, amikacin; GM, gentamycin*.

### The combined therapy of COG1410 and polymyxin B is capable of rescuing *C. elegans* infected by *A. baumannii*

**Figure 8:**
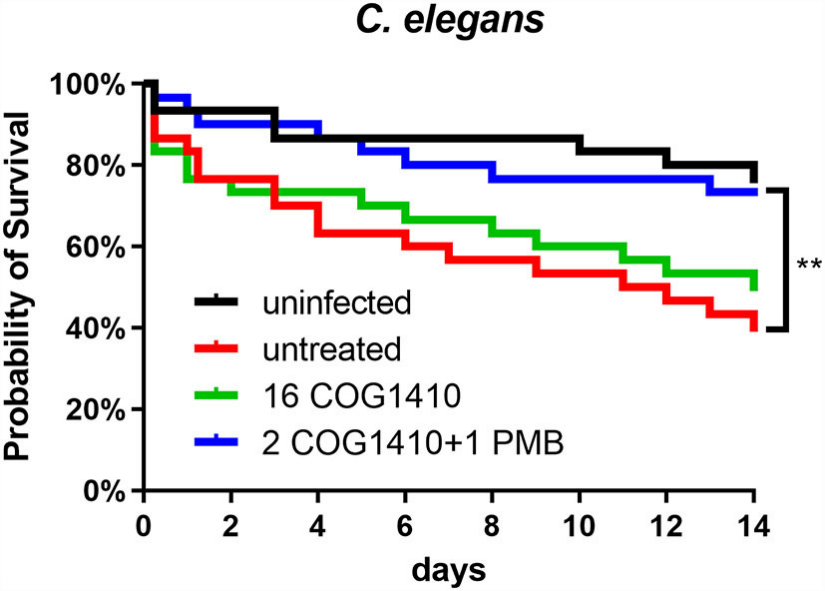
The combined therapy of COG1410 and polymyxin B rescued infected nematodes. The cultures of *C. elegans* were pre-infected with *A. baumannii* YQ4 and transferred to an NGM plate supplemented with 16 μg/ml COG1410 or 2 μg/ml COG1410 and 1 μg/ml polymyxin B. The dead nematodes were counted every day for 2 weeks. The survival curve was analyzed by Kaplan–Meier method, and the statistical significance was analyzed by the log-rank test. **p < 0.01.

To address the effectiveness *in vivo*, we decided to utilize *C. elegans* to develop an infection model. The L4 nematodes were pre-infected in M9 buffer and transferred to an NGM agar plate supplemented with or without COG1410. To increase the pathogenesis of *A. baumannii*, 10 μM FeCl_3_ was added as previously suggested (Vallejo et al., 2015). Compared with the untreated group, 16 μg/ml of COG1410 alleviated the death of infected nematodes but not statistically significantly. To our interest, the combination therapy of COG1410 (2 μg/ml) and polymyxin B (1 μg/ml) significantly rescued the pre-infected nematodes and even elongated their lives (Figure 8).

## Discussion

In the present study, we evaluated the antibacterial efficacy of ApoE mimetic peptide COG1410 and revealed its mechanism of action. COG1410 exhibited a broad antibacterial spectrum and potent bactericidal activity, especially against the PDR *A. baumannii* strains. Similar to most other cationic AMPs, COG1410 took effect very rapidly *in vitro*. The 1× MIC of COG1410 reduced 3 log CFU/ml of *A. baumanii* even within 5 min, which was much faster than polymyxin B and equivalent to that of the promising anti-*A. baumannii* AMP, ZY4, which is a cathelicidin-derived peptide and completely kills MDR *A. baumannii* within 30 min at 1× MIC (Mwangi et al., 2019). Besides the planktonic cells, COG1410 could inhibit the biofilm formation and eradicate the mature biofilm. *A. baumannii* is one of the major biofilm-producing bacteria. Just because of its ability to form biofilms, it could easily survive and transfer to the hospital environment (Colquhoun and Rather, 2020). Therefore, COG1410 is a promising candidate for the development of new antimicrobials.

The major challenge for the therapeutic application of AMP is the degradation or inactivation in plasma (Dijksteel et al., 2021). The bactericidal efficacy of COG1410 was further assessed in plasma. The LC_99.9_ values of COG1410 in PBS and 50% pooled plasma were 1.4 and 5.6 μM, respectively. This was even better than LL-37-derived AMP, SAAP-148, of which the corresponding LC_99.9_ values were 1.6 and 12.8 μM, against *A. baumannii*, respectively (De Breij et al., 2018). The stability assay showed that COG1410 was very stable in plasma. Based on the inhibition diameter, COG1410 does not seem to be significantly degraded in 100% human plasma within 2 h. Even after 10 h, more than 80% of the activity was maintained. This excellent stability might be attributed to the substitution of unnatural amino acid, aminoisobutyric acid, in COG1410. A recent study showed that the incorporation of a single Aib residue at the N-terminus of AMP provided remarkably enhanced plasma stability and improved activity *in vivo* (Lu et al., 2020). To be consistent with this, COG1410 has been applied *via* intravenous injection. In the murine model of TBI, a single intravenous injection of COG1410 significantly improved vestibulomotor function, spatial learning, and memory (Laskowitz et al., 2007). A similar effect was observed in the rat model of focal brain ischemia and mice model of traumatic optic nerve injury (Tukhovskaya et al., 2009; Kuai et al., 2019). These data show that COG1410 is a potential AMP for systemic infection.

Most cationic AMPs directly target the cell membrane, causing pore formation and final cell lysis (Zhang and Gallo, 2016). In our study, we observed that treatment with polymyxin B leads to the collapse of the cell wall and the release of cytoplasmic content. However, the cells treated with COG1410 seemed to be still intact without pore formation. The cause of death was probably due to the separation between the inner membrane and the cell wall. A similar foamy disintegrating membrane was observed in *A. baumannii* cells treated with AMP Cec4 (Peng et al., 2019). We did observe the significant ATP leak of cells exposed to COG1410, which indicated that the cell membrane was disrupted or permeabilized. Consistently, the fluorescent dye staining assay also confirmed this conclusion. On the other hand, we observed that COG1410 could cross the cell membrane and enter the cytoplasm of *A. baumannii*. RNA-seq analysis revealed that sub-MIC of COG1410 significantly affected the minor part of genes of *A. baumannii* in the minimal medium, while the genes involved in the oxidative and reductive biological process were remarkably enriched. DCFH-DA probe also detected 30% more ROS after COG1410 treatment for 20 min. These data suggested that COG1410 might induce oxidative stress in *A. baumannii*, which was similar to LL-37 (Choi et al., 2017). Meanwhile, COG1410 bound with DNA nonspecifically. It has been suggested that membrane disruption and DNA binding were two hits of the AMP, NK18 (Yan et al., 2013). However, the confusing point was that COG1410 was also able to enter the cytoplasm of *P. aeruginosa, S. aureus*, and *E. faecium*, but these bacteria were not sensitive. It seemed that DNA binding might not play a key role. The mechanism involved in the bactericidal spectrum of COG1410 was worthy to study further. Taken together, COG1410 inhibited bacterial growth by two mechanisms: disruption of the integrity of cell membrane and induction of oxidative stress.

As a class of drugs, cationic AMPs are particularly troublesome with regard to cytotoxicity. We observed that 128 μg/ml (8× MIC in LB) concentration of COG1410 only leads to <5% hemolysis. The EC_50_ of COG1410 was 58.9 μg/ml for the L02 cell. Fortunately, the LC_99.9_ was 2 and 8 μg/ml in PBS and 50% plasma, respectively. Furthermore, there was significant synergistic interaction between COG1410 and polymyxin B, where the working concentration was reduced to 2 μg/ml for COG1410 and 1 μg/ml for polymyxin B. We noticed that the bactericidal effect of COG1410 did not depend on the electrostatic interaction with LPS, since modification of LPS did not inhibit the activity of COG1410. This feature was in contrast with polymyxin B and LL-37, whose initial step in the attacking is binding with LPS (Sochacki et al., 2011). Polymyxin B binds with the lipid A portion of the LPS in Gram-negative bacteria, replacing cationic ions such as Ca^2+^ and Mg^2+^, which destabilizes the LPS layer and the membrane (Abdul Rahim et al., 2015). In fact, Polymyxin B also showed synergistic interaction with many short cationic AMPs (Ruden et al., 2019). Since combination drug therapy is frequently used in medical practice, especially in the intensive care units, the synergy with polymyxin B makes COG1410 possible for clinical treatment in the future.

*Caenorhabditis elegans* is a good non-mammalian animal model for screening anti-infective compounds (Peterson and Pukkila-Worley, 2018). This worm could be infected by a wide range of human bacterial pathogens, including *A. baumannii*. Compared with other human pathogens, like *Pseudomonas aeruginosa* and *Staphylococcus aureus*, the killing rate of *A. baumannii* is much slower, indicating a much lower level of virulence against the nematodes. An alternative way is to use an immunocompromised *C. elegans* mutant with glp-4(bn2ts)/sek-1(km4) mutation, which is more sensitive than the wild-type N2 to bacterial killing. Also, it has been reported that the addition of FeCl_3_ increases the killing rate in the liquid medium (Ouyang et al., 2017). In our study, we established the infection model with *A. baumannii* with FeCl_3_ and then tried to treat these infected nematodes. The survival curve showed that the combination therapy with COG1410 and polymyxin B did work to save the worms. However, since a slow rate of killing is observed even with FeCl_3_, the missed worms and dead ones with starvation increased soon over time, which seriously interrupted observation and comparison. Therefore, to clearly display the difference in life span between treated and untreated worms, we only exhibited data for 2 weeks.

In conclusion, our study revealed the potent antibacterial capability of the ApoE mimetic peptide, COG1410. The major bactericidal mechanism of COG1410 was to disrupt cell membrane integrity and induce oxidative stress. COG1410 displayed strong bacterial killing, high stability in human plasma, and a low propensity for resistance development. The synergistic interaction between COG1410 and polymyxin B reduces the working concentration of COG1410 and avoids the risk of cell toxicity. Considering that it simultaneously has neuroprotective, anti-inflammation, and antibacterial effects, COG1410 represents a promising therapeutic candidate to overcome the crisis of PDR *A. baumannii*.

## Data availability statement

The data presented in the study are deposited in the GenBank and SRA database with accession numbers CP053033 and PRJNA833738, respectively.

## Ethics statement

The studies involving human participants were reviewed and approved by Institutional Review Boards of the Second Hospital of Nanjing (2021-LY-kt060). The patients/participants provided their written informed consent to participate in this study.

## Author contributions

BW measured the bactericidal spectrum and activity *in vitro*. F-WZ and W-XW analyzed the mechanisms of action and activity *in vivo*. BW, F-WZ, and W-XW drafted the manuscript. Y-YZ collected data from the CLSM. S-YS conducted RNA-seq and data analysis. J-HY conducted the hemolysis and cell toxicity test. MV and GL synthesized the peptide. RM performed SEM and TEM. SW measured the ROS levels and ATP leak. ZH contributed ideas to the project and analyzed the data. WC conceived the study, directed the experiments, and revised the manuscript. All authors approved the submission.

## Funding

This work was supported by the General Foundation of Jiangsu Provincial Health Committee (M2020019) and a grant from the Nanjing Medical Science and Technique Development Foundation (ZKX21037).

## Conflict of interest

Authors MV and GL were employed by Cognosci, Inc. Author RM was employed by Shanghai Nanoport, Thermofisher Scientific. The remaining authors declare that the research was conducted in the absence of any commercial or financial relationships that could be construed as a potential conflict of interest.

## Publisher's note

All claims expressed in this article are solely those of the authors and do not necessarily represent those of their affiliated organizations, or those of the publisher, the editors and the reviewers. Any product that may be evaluated in this article, or claim that may be made by its manufacturer, is not guaranteed or endorsed by the publisher.

## Supplementary material

Supplementary Figure S1 COG1410 maintained stability in human plasma. About 10 mg/ml of COG1410 was incubated with 100% human plasma at 37°C and sampled at different time points. The stability of COG1410 was determined by evaluating the antibacterial activity against PDR *A. baumannii* YQ4 through the disk diffusion assay. The diameters of inhibition were measured. Three independent experiments were performed and data were represented as mean ± SD. The statistical significance between initial and different points was analyzed by Student's *t*-test. ^*^ indicated *p* < 0.05; ^***^ indicated *p* < 0.001.

Supplementary Figure S2 The membrane integrity was compromised in the presence of COG1410. Fluorescence microscopy images of PDR *A. baumannii* YQ4 treated with 1× MIC of COG1410 or 5× MIC of polymyxin B at 37°C for 30 min and stained with SYTO-9 and PI. Scale bar: 15 μm.

Supplementary Figure S3 DNA-binding activity of COG1410. Migration of pUC18 was retarded in 1.5% agarose gel electrophoresis by COG1410. The gel was visualized after Genered nucleic acid dye staining and UV irradiation. The numbers above the lanes represented the concentration of COG1410 (1 × MIC).

Supplementary Figure S4 DEGs in GO level 2.

Supplementary Figure S5 COG1410 showed low propensity to induce resistance. PDR *A. baumannii* strain YQ4 was serially passaged in the presence of sub-MIC COG1410. Polymyxin B acted as a positive control. The MIC change was determined by a microdilution assay.

Supplementary Table S1 Antibacterial effect of ApoE-derived peptides.

Supplementary Table S2 Determination of bactericidal effect of COG1410 against clinically collected *A. baumannii* strains. MIC was measured in LB broth by microdilution method.

Supplementary Table S3 Differential expressed genes of *A. baumannii* treated by sub-MIC COG1410.
