# Supplementary figures and images for: Apolipoprotein E mimetic peptide COG1410 combats pandrug-resistant Acinetobacter baumannii
Source: Front Microbiol. 2022 Aug 23;13:934765. doi: 10.3389/fmicb.2022.934765 (PMC9445589; doi:10.3389/fmicb.2022.934765)

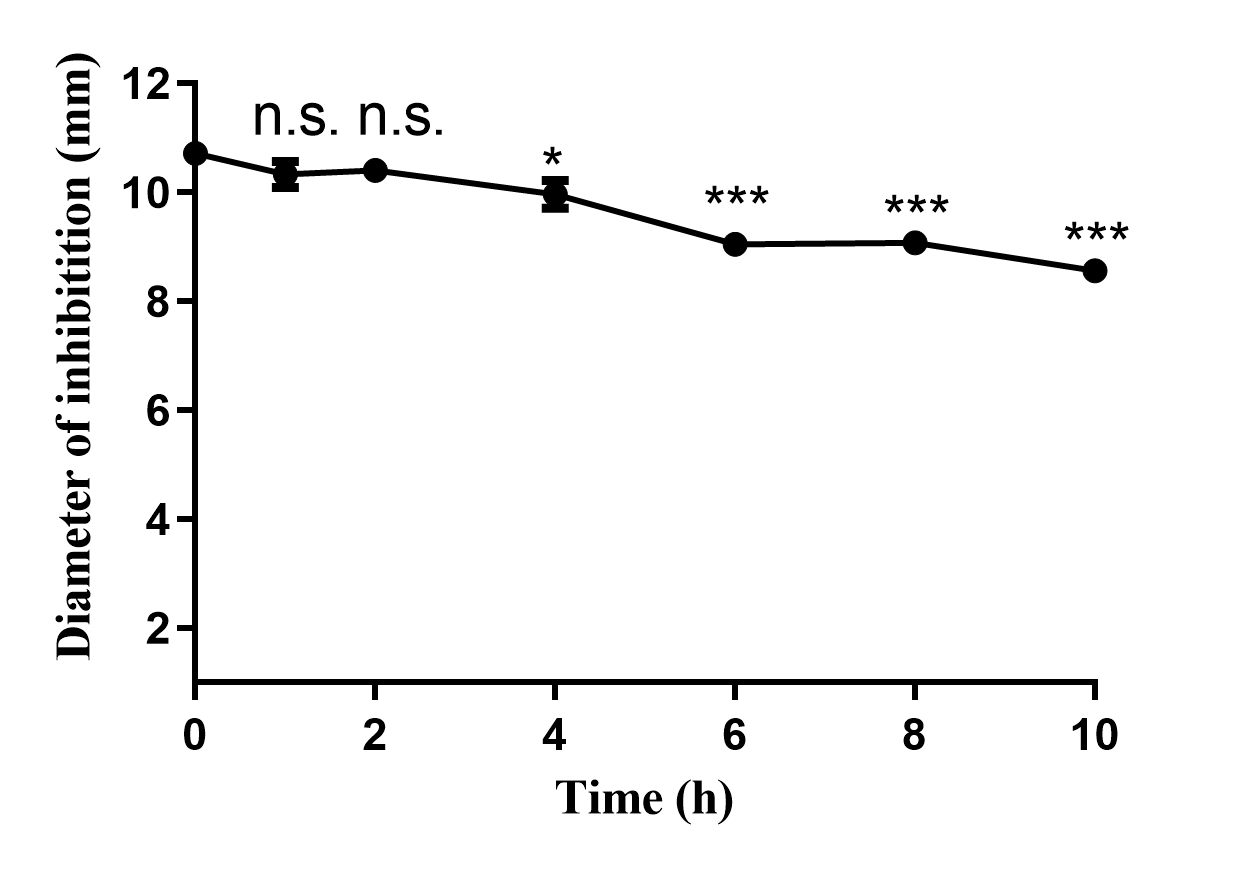

Supplement: Supplementary Figure S1 — COG1410 maintained stability in human plasma. About 10 mg/ml of COG1410 was incubated with 100% human plasma at 37°C and sampled at different time points. The stability of COG1410 was determined by evaluating the antibacterial activity against PDR A. baumannii YQ4 through the disk diffusion assay. The diameters of inhibition were measured. Three independent experiments were performed and data were represented as mean ± SD. The statistical significance between initial and different points was analyzed by Student's t-test. * indicated p < 0.05; *** indicated p < 0.001. [file Image_1.TIF]

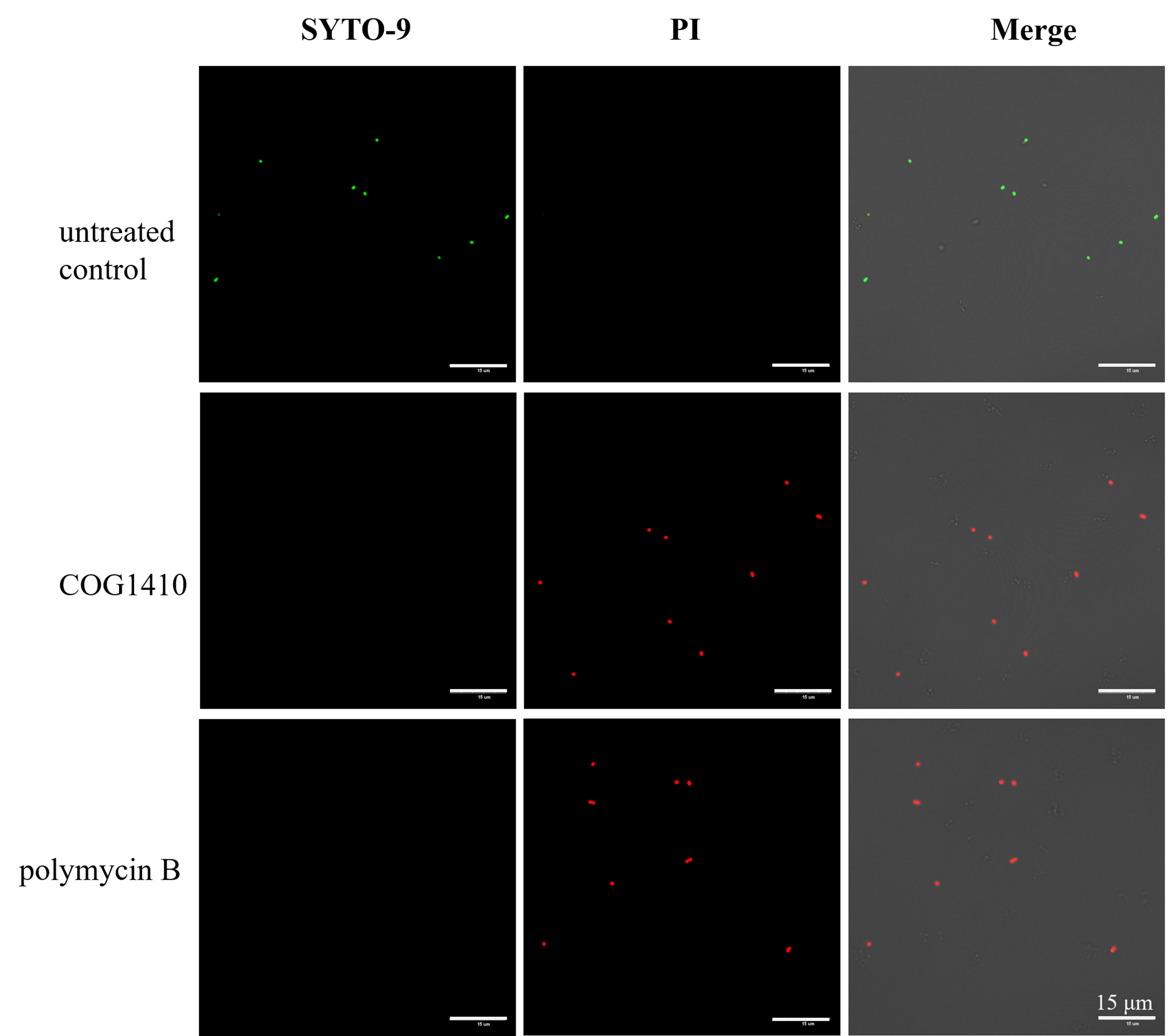

Supplement: Supplementary Figure S2 — The membrane integrity was compromised in the presence of COG1410. Fluorescence microscopy images of PDR A. baumannii YQ4 treated with 1× MIC of COG1410 or 5× MIC of polymyxin B at 37°C for 30 min and stained with SYTO-9 and PI. Scale bar: 15 μm. [file Image_2.TIF]

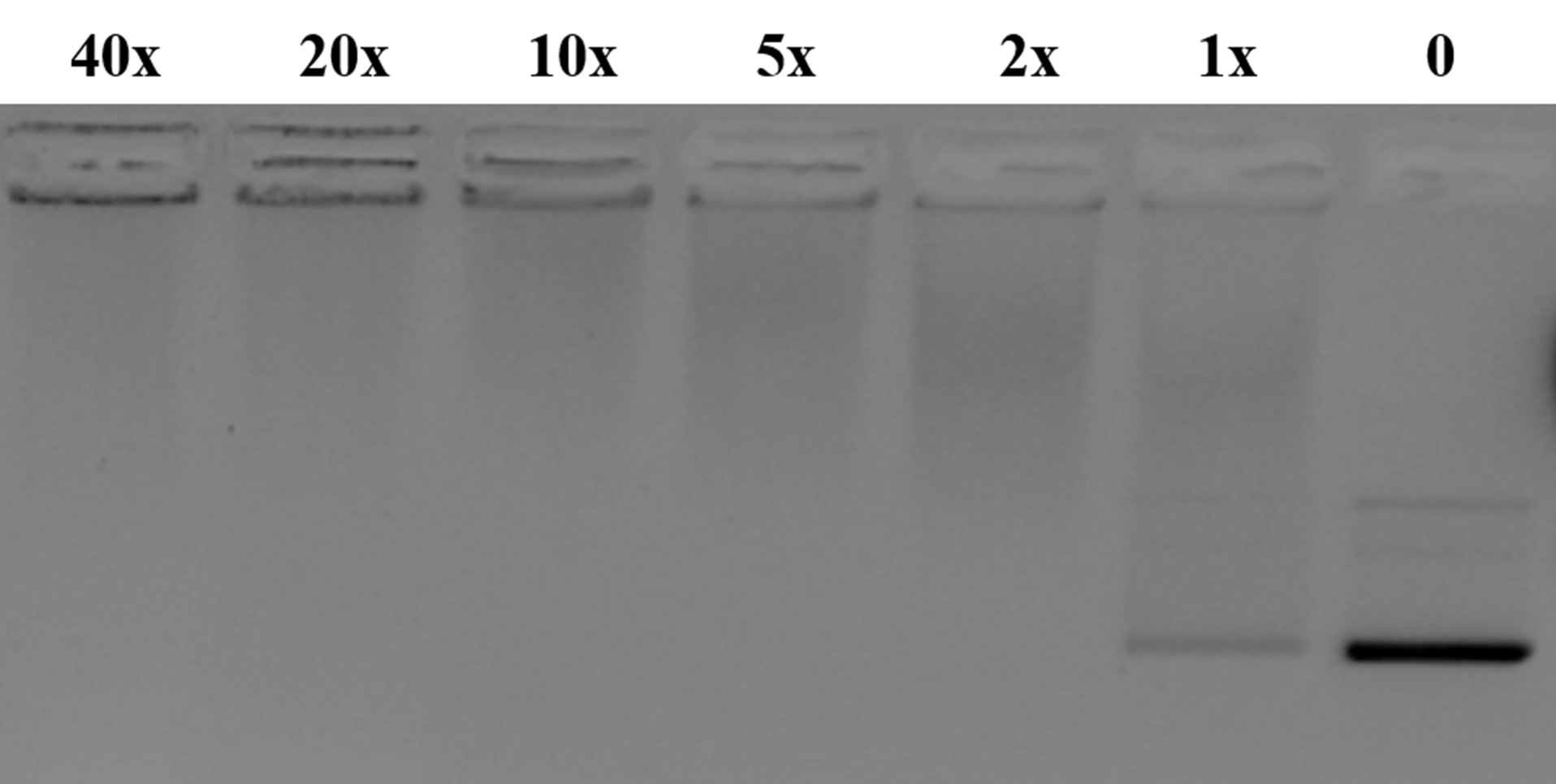

Supplement: Supplementary Figure S3 — DNA-binding activity of COG1410. Migration of pUC18 was retarded in 1.5% agarose gel electrophoresis by COG1410. The gel was visualized after Genered nucleic acid dye staining and UV irradiation. The numbers above the lanes represented the concentration of COG1410 (1 × MIC). [file Image_3.TIF]

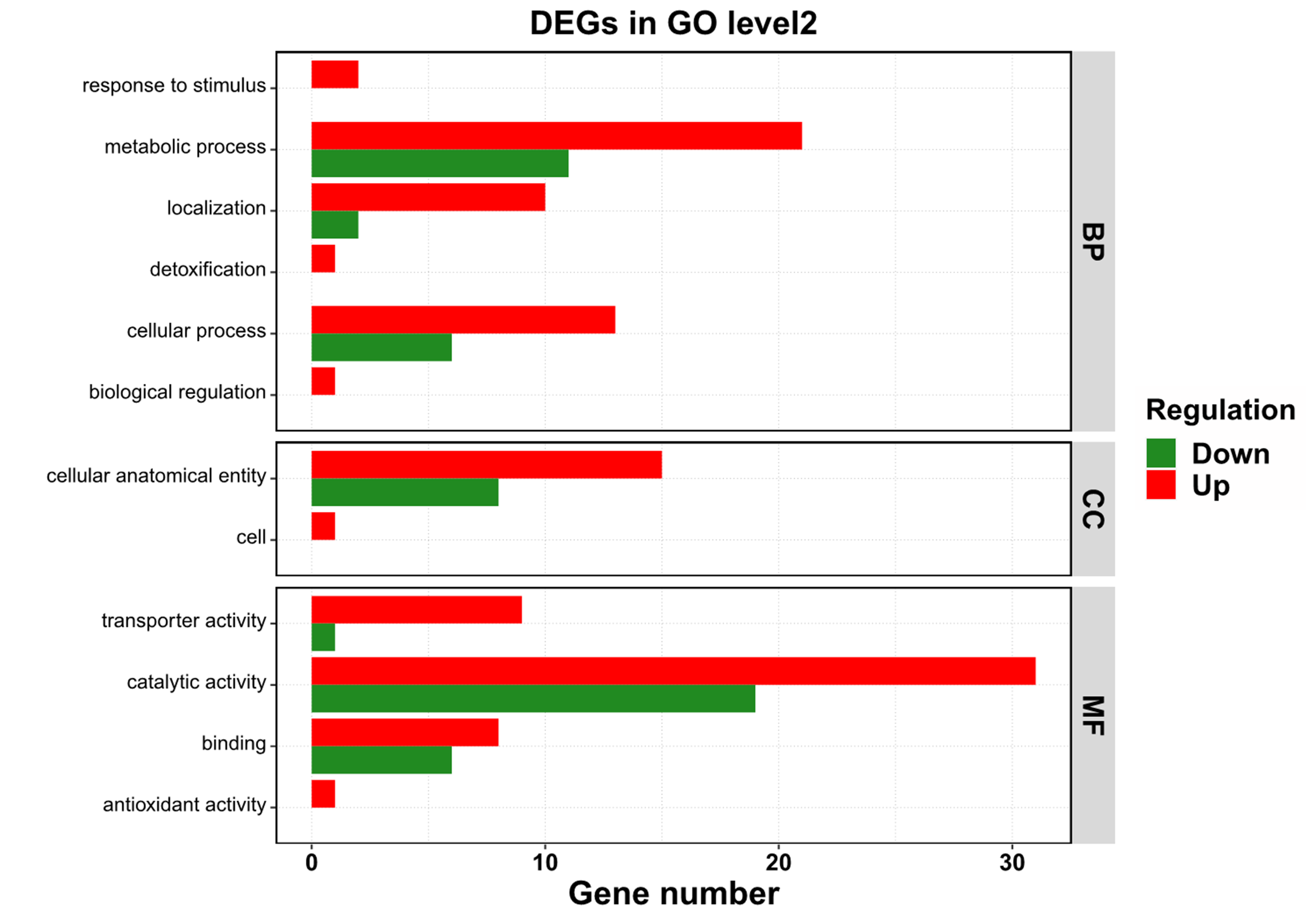

Supplement: Supplementary Figure S4 — DEGs in GO level 2. [file Image_4.TIF]

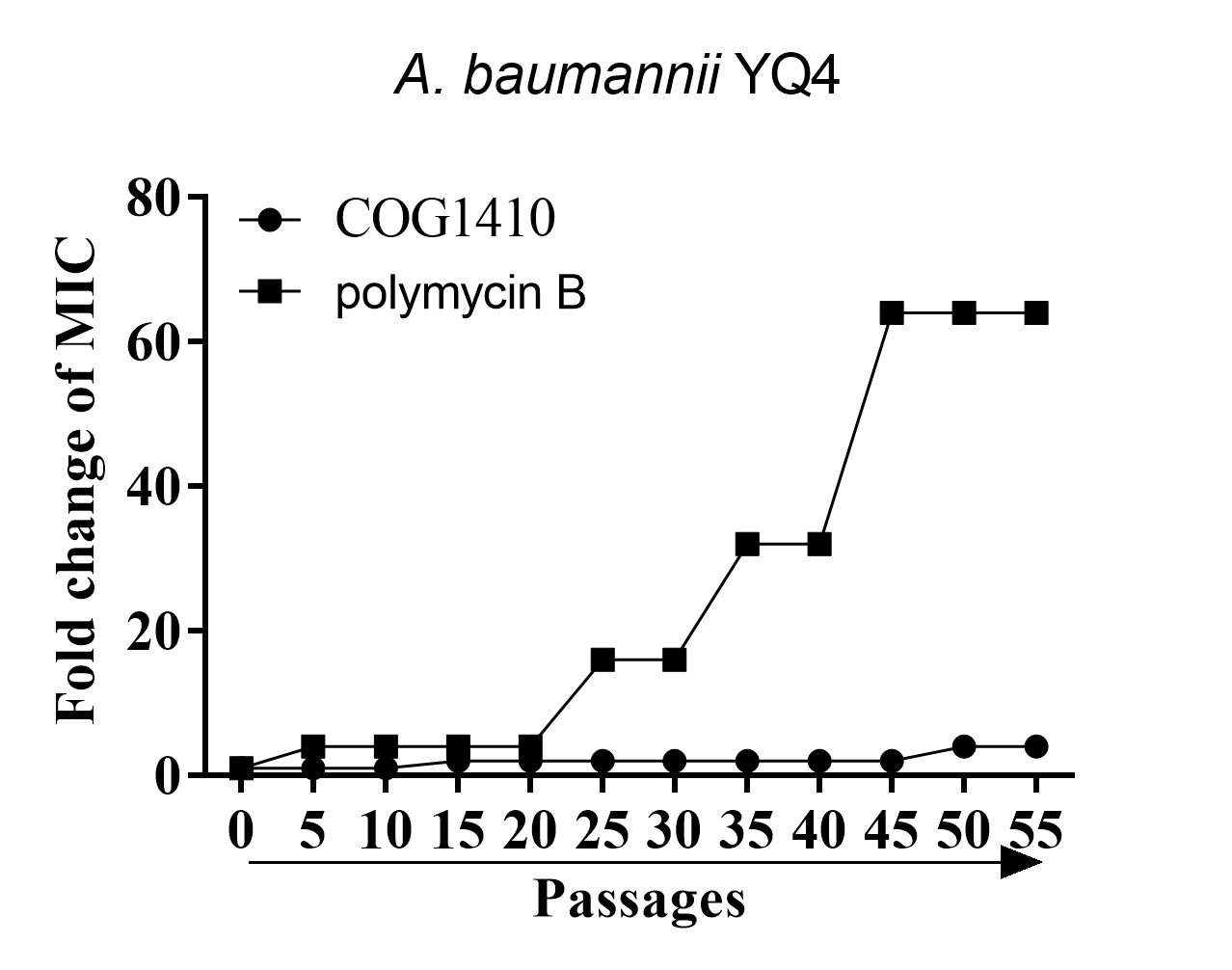

Supplement: Supplementary Figure S5 — COG1410 showed low propensity to induce resistance. PDR A. baumannii strain YQ4 was serially passaged in the presence of sub-MIC COG1410. Polymyxin B acted as a positive control. The MIC change was determined by a microdilution assay. [file Image_5.TIF]
