# Supplementary material for: Apolipoprotein E mimetic peptide COG1410 combats pandrug-resistant Acinetobacter baumannii
Source: Front Microbiol. 2022 Aug 23;13:934765. doi: 10.3389/fmicb.2022.934765 (PMC9445589; doi:10.3389/fmicb.2022.934765)
Supplement: Supplementary Table S1 — Antibacterial effect of ApoE-derived peptides. [file Table_1.DOCX]

**Table S1. Antibacterial effect of ApoE-derived peptides.**

| **Peptide name** | **Locations in ApoE** | **Amino acid sequence** | **MIC (μM)** | | | | | | |
| --- | --- | --- | --- | --- | --- | --- | --- | --- | --- |
|  |  |  | **Pa** | **Ab** | **Kp** | **Ef** | **Ec** | **Sa** | **References** |
| ApoE_23_ | 141-148+135-149 | LRKLRKRLVRLASHLRKLRKRLL | 2.7 | 2.0 | - | void | 2.0 | 5.4 | (1) |
| ApoE_30_ | 133-162 | LRVRLASHLRKLRKRLLRDADDLQKRLAVY | 8.1 | - | - | - | 1.3 | 16.2 | (2) |
| ApoE_p_ | 141-149+141-149 | LRKLRKRLLLRKLRKRLL | 5 | - | - | - | - | 35 | (1, 3) |
| ApoE_p-W_ | 141-149+141-149 | WRKWRKRWWWRKWRKRWW | - |  | <3.3 | - | - | <3.3 | (4) |
| sApoE_133-150_ | 133-150 | LRVRLASHLRKLRKRLLR | 25/50 | - | 6.25 | - | 6.25/12.5 | 3.12 | (5) |
| COG133 | 133-149 | LRVRLASHLRKLRKRLL | 25/50 | - | 3.12 | - | 6.25/12.5 | void | (5) |
| ApoE_141-149_ | 141-149 | LRKLRKRLL | 30 | - | - | - | - | void | (3) |

Note：Pa, *Pseudomonas aeruginosa*; Sa, *Staphylococcus aureus*; Ab, *Acinetobacter baumannii*; Kp, *Klebsiella pneumoniae*; Ef, *Enterococcus faecium*; Ec, *Escherichia coli*. “-” indicates undetermined. “void” indicates that the MIC is more than 100 μM.

**References**

1. Wang CQ, Yang CS, Yang Y, Pan F, He LY, Wang AM. An apolipoprotein E mimetic peptide with activities against multidrug-resistant bacteria and immunomodulatory effects. Journal of peptide science : an official publication of the European Peptide Society. 2013;19(12):745-50.

2. Azuma M, Kojimab T, Yokoyama I, Tajiri H, Yoshikawa K, Saga S, et al. A synthetic peptide of human apoprotein E with antibacterial activity. Peptides. 2000;21(3):327-30.

3. Dobson CB, Sales SD, Hoggard P, Wozniak MA, Crutcher KA. The receptor-binding region of human apolipoprotein E has direct anti-infective activity. The Journal of infectious diseases. 2006;193(3):442-50.

4. Forbes S, McBain AJ, Felton-Smith S, Jowitt TA, Birchenough HL, Dobson CB. Comparative surface antimicrobial properties of synthetic biocides and novel human apolipoprotein E derived antimicrobial peptides. Biomaterials. 2013;34(22):5453-64.

5. Pane K, Sgambati V, Zanfardino A, Smaldone G, Cafaro V, Angrisano T, et al. A new cryptic cationic antimicrobial peptide from human apolipoprotein E with antibacterial activity and immunomodulatory effects on human cells. The FEBS Journal. 2016;283(11):2115-31.
