# Supplementary material for: Apolipoprotein E mimetic peptide COG1410 combats pandrug-resistant Acinetobacter baumannii
Source: Front Microbiol. 2022 Aug 23;13:934765. doi: 10.3389/fmicb.2022.934765 (PMC9445589; doi:10.3389/fmicb.2022.934765)
Supplement: Supplementary Table S2 — Determination of bactericidal effect of COG1410 against clinically collected A. baumannii strains. MIC was measured in LB broth by microdilution method. [file Table_2.docx]

**TAble S2. Determination of bactericidal effect of COG1410 against clinically collected *A. baumannii* strains.** MIC was measured in LB broth by microdilution method.

| **No.** | **Strain name** | **Source** | **MIC** |
| --- | --- | --- | --- |
| Ab3-1 | MDR-Ab | hydrothorax and ascite | **32** |
| Ab3-2 | MDR-Ab | sputum | 16 |
| Ab3-3 | MDR-Ab | sputum | 16 |
| Ab3-8 | MDR-Ab | sputum | 16 |
| Ab3-11 | Ab | sputum | 16 |
| Ab3-12 | Ab | hydrothorax | 16 |
| Ab3-14 | Ab | sputum | 16 |
| Ab3-16 | MDR-Ab | sputum | 16 |
| Ab3-21 | MDR-Ab | sputum | 16 |
| Ab3-27 | MDR-Ab | catheter | 16 |
| Ab3-36 | MDR-Ab | urine | 16 |
| Ab3-37 | MDR-Ab | sputum | 16 |
| Ab3-39 | MDR-Ab | sputum | 16 |
| Ab3-40 | MDR-Ab | peripheral blood | 16 |
| Ab3-41 | Ab | sputum | 16 |
| Ab3-42 | Ab | sputum | 16 |
| Ab3-44 | Ab | hydrothorax | 16 |
| Ab3-45 | MDR-Ab | sputum | 16 |
| Ab3-46 | Ab | hydrothorax | 16 |
| Ab3-47 | Ab | sputum | 16 |
| Ab3-50 | Ab | blood | 16 |
| Ab3-51 | MDR-Ab | sputum | 16 |
| Ab3-52 | MDR-Ab | sputum | 16 |
| Ab3-53 | MDR-Ab | sputum | 16 |
| Ab3-54 | MDR-Ab | sputum | 16 |
| Ab3-55 | MDR-Ab | urine | 16 |
| Ab3-60 | Ab | sputum | 16 |
| Ab3-62 | Ab | sputum | 16 |
| Ab3-63 | Ab | sputum | 16 |
| Ab3-65 | Ab | sputum | 16 |
| Ab3-68 | MDR-Ab | sputum | 16 |
| Ab3-69 | CR-Ab | sputum | 16 |
| Ab3-70 | Ab | sputum | 16 |
| Ab3-71 | MDR-Ab | sputum | 16 |
| Ab3-72 | Ab | blood | 16 |
| Ab3-73 | MDR-Ab | sputum | 16 |
| Ab3-110 | Ab | sputum | 16 |
| Ab10-29 | MDR-Ab | sputum | 16 |
| Ab10-35 | MDR-Ab | sputum | 16 |
| Ab10-36 | Ab | sputum | 16 |
| Ab10-38 | MDR-Ab | sputum | 16 |
| Ab10-39 | MDR-Ab | sputum | 16 |
| Ab10-44 | MDR-Ab | sputum | 16 |
| Ab10-47 | MDR-Ab | sputum | 16 |
| Ab10-79 | MDR-Ab | sputum | 16 |
| Ab11-21 | MDR-Ab | sputum | 16 |
| Ab11-23 | MDR-Ab | bile | 16 |
| Ab11-24 | MDR-Ab | sputum | 16 |
| Ab11-35 | Ab | whole blood | 16 |
| Ab11-42 | Ab | whole blood | 16 |
| Ab11-44 | MDR-Ab | sputum | 16 |
| Ab11-46 | MDR-Ab | sputum | 16 |
| Ab11-49 | Ab | sputum | 16 |
| Ab11-52 | Ab | sputum | 16 |
| Ab11-58 | MDR-Ab | whole blood | 16 |
| Ab11-59 | MDR-Ab | sputum | 16 |
| Ab11-60 | MDR-Ab | sputum | 16 |
| Ab11-66 | MDR-Ab | sputum | 16 |
| Ab11-67 | MDR-Ab | sputum | 16 |
| Ab11-71 | MDR-Ab | whole blood | 16 |
| Ab11-74 | MDR-Ab | urine | 16 |
| Ab11-75 | MDR-Ab | sputum | 16 |
| Ab11-78 | Ab | sputum | 16 |
| Ab11-84 | MDR-Ab | sputum | **64** |
| Ab11-85 | MDR-Ab | sputum | 16 |
| Ab11-86 | MDR-Ab | sputum | 16 |
| Ab11-87 | MDR-Ab | ascites | 16 |
| Ab11-88 | MDR-Ab | sputum | 16 |
| Ab11-89 | MDR-Ab | sputum | 16 |
| Ab11-96 | Ab | whole blood | **32** |
| Ab14-1 | CR-Ab | sputum | 16 |
| Ab14-4 | MDR-Ab | sputum | 16 |
| Ab14-7 | MDR-Ab | sputum | 16 |
| Ab14-10 | MDR-Ab | whole blood | 16 |
| Ab14-13 | MDR-Ab | hydrothorax | 16 |
| Ab14-15 | MDR-Ab | cerebrospinal fluid | 16 |
| Ab14-16 | MDR-Ab | cerebrospinal fluid | 16 |
| Ab14-17 | MDR-Ab | sputum | 16 |
| Ab14-18 | MDR-Ab | sputum | 16 |
| Ab14-21 | MDR-Ab | sputum | 16 |
| Ab14-24 | MDR-Ab | sputum | 16 |
| Ab14-26 | MDR-Ab | sputum | 16 |
| Ab14-27 | MDR-Ab | sputum | 16 |
| Ab14-28 | MDR-Ab | sputum | 16 |
| Ab14-29 | MDR-Ab | sputum | 16 |
| Ab14-35 | MDR-Ab | sputum | 16 |
| Ab14-36 | MDR-Ab | sputum | 16 |
| Ab14-38 | MDR-Ab | sputum | 16 |
| Ab14-39 | MDR-Ab | sputum | 16 |
| Ab14-40 | MDR-Ab | sputum | 16 |
| Ab14-42 | MDR-Ab | sputum | 16 |
| Ab14-50 | CR-Ab | sputum | 16 |
| Ab14-51 | MDR-Ab | urine | 16 |
| Ab14-53 | Ab | whole blood | 16 |
| Ab14-64 | Ab | sputum | 16 |
| Ab14-71 | MDR-Ab | ascites | 16 |
| Ab14-72 | MDR-Ab | sputum | 16 |
| Ab14-73 | Ab | sputum | 16 |
| Ab14-74 | MDR-Ab | sputum | 16 |
| Ab14-75 | MDR-Ab | sputum | 16 |
| Ab14-76 | MDR-Ab | sputum | 16 |
| Ab14-77 | MDR-Ab | sputum | 16 |
| Ab14-78 | MDR-Ab | sputum | 16 |
| Ab14-79 | MDR-Ab | sputum | 16 |
| Ab14-80 | MDR-Ab | sputum | 16 |
| Ab14-81 | CR-Ab | sputum | 16 |
| Ab14-82 | CR-Ab | sputum | 16 |

Note: MDR, multiple-drug resistance; CR, carbapenemase resistance
